# Genomic diversity and meiotic recombination among isolates of the biotech yeast *Komagataella phaffii* (*Pichia pastoris*)

**DOI:** 10.1186/s12934-019-1260-4

**Published:** 2019-12-04

**Authors:** Stephanie Braun-Galleani, Julie A. Dias, Aisling Y. Coughlan, Adam P. Ryan, Kevin P. Byrne, Kenneth H. Wolfe

**Affiliations:** 1 UCD Conway Institute, School of Medicine, University College Dublin, Dublin, Ireland; 2 Department of Mathematics and Statistics, McGill University, Montreal, QC Canada

**Keywords:** Genetic diversity, Recombination, Evolution, Yeasts

## Abstract

**Background:**

*Komagataella phaffii* is a yeast widely used in the pharmaceutical and biotechnology industries, and is one of the two species that were previously called *Pichia pastoris*. However, almost all laboratory work on *K. phaffii* has utilized strains derived from a single natural isolate, CBS7435. There is little information about the sequence diversity of *K.* *phaffii* or the genetic properties of this species.

**Results:**

We sequenced the genomes of all the known isolates of *K. phaffii.* We made a genetic cross between derivatives of two isolates that differ at 44,000 single nucleotide polymorphism sites, and used this cross to analyze the rate and landscape of meiotic recombination. We conducted tetrad analysis by making use of the property that *K.* *phaffii* haploids do not mate in rich media, which enabled us to isolate and sequence the four types of haploid cell that are present in the colony that forms when a tetra-type ascus germinates.

**Conclusions:**

We found that only four distinct natural isolates of *K. phaffii* exist in public yeast culture collections. The meiotic recombination rate in *K. phaffii* is approximately 3.5 times lower than in *Saccharomyces cerevisiae*, with an average of 25 crossovers per meiosis. Recombination is suppressed, and genetic diversity among natural isolates is low, in a region around centromeres that is much larger than the centromeres themselves. Our work lays a foundation for future quantitative trait locus analysis in *K. phaffii.*

## Background

*Komagataella phaffii* is the most widely used yeast species for production of heterologous proteins, such as the expression of antibody fragments for the pharmaceutical industry. It has several advantages over *Saccharomyces cerevisiae* as a cell factory, including thermotolerance, respiratory growth to very high cell densities, and the ability to express foreign proteins at high levels from either constitutive promoters or its inducible methanol oxidase promoter [1–4]. *K. phaffii* is better known under its previous name *Pichia pastoris*, but the name ‘*P.* *pastoris*’ was discontinued in 2009 after it was discovered to have been used for heterogeneous strains that actually belong to two separate species, which were renamed *K. phaffii* and *K.* *pastoris* [5]. Their genomes differ by approximately 10% DNA sequence divergence and two reciprocal translocations [6]. Phylogenetically, *Komagataella* species are members of the methylotrophic yeasts clade (family Pichiaceae) and are only distantly related to better-known yeasts such as *S.* *cerevisiae* and *Candida albicans* [7].

Almost all research on *K. phaffii* has been done using the genetic background of strain CBS7435 (synonymous with NRRL Y-11430) [4]. The origin of this strain has been unclear because it was deposited in the CBS and NRRL culture collections in connection with a US patent granted to Phillips Petroleum (see discussion in [5]), but we show here that CBS7435 is identical to the type strain of *K.* *phaffii*, which was isolated from an oak tree. The widely-used *K. phaffii* strains GS115 and X-33 are derivatives of CBS7435 and are components of a commercial protein expression kit marketed by Invitrogen/Life Technologies/Thermo Fisher. GS115 was made from CBS7435 by random mutagenesis with nitrosoguanidine and includes a *his4* mutation among a few dozen point mutations [6, 8]. X-33 is a derivative of GS115 in which the *HIS4* gene was reverted to wildtype by site-directed mutagenesis [9].

The public yeast culture collections include a few natural isolates of *K. phaffii*, but these have received little attention. Most of them were isolated from exudates (slime fluxes) on trees. The genetic diversity that is naturally present in populations of *K.* *phaffii* could potentially be used to improve its performance in biotechnological applications. In principle, beneficial alleles from natural isolates could be introduced into biotech strains by breeding. As a first step towards this goal, in this study we surveyed the nucleotide sequence diversity that is present in all six isolates of *K. phaffii* that are available from public culture collections.

Although techniques for inserting foreign genes into the *K. phaffii* genome and controlling their expression are well established, other aspects of the genetics and life cycle of this yeast are much less studied [10]. *K. phaffii* has four chromosomes and grows primarily as a haploid. Mating only occurs when induced by nitrogen depletion [10–12]. Zygotes usually sporulate immediately after mating, but in crosses between haploids carrying auxotrophic markers, the diploid progeny can be maintained by transferring them to nitrogen-replete media and selecting for prototrophy [11]. Mating occurs between *MAT*a and *MAT*α cells, and haploids can switch their mating type by inverting a 138-kb section of chromosome 4 [12, 13]. The recent development of stable heterothallic strains of *K. phaffii* makes it possible to carry out controlled genetic crosses without requiring selectable markers [13, 14].

**Fig. 1:**
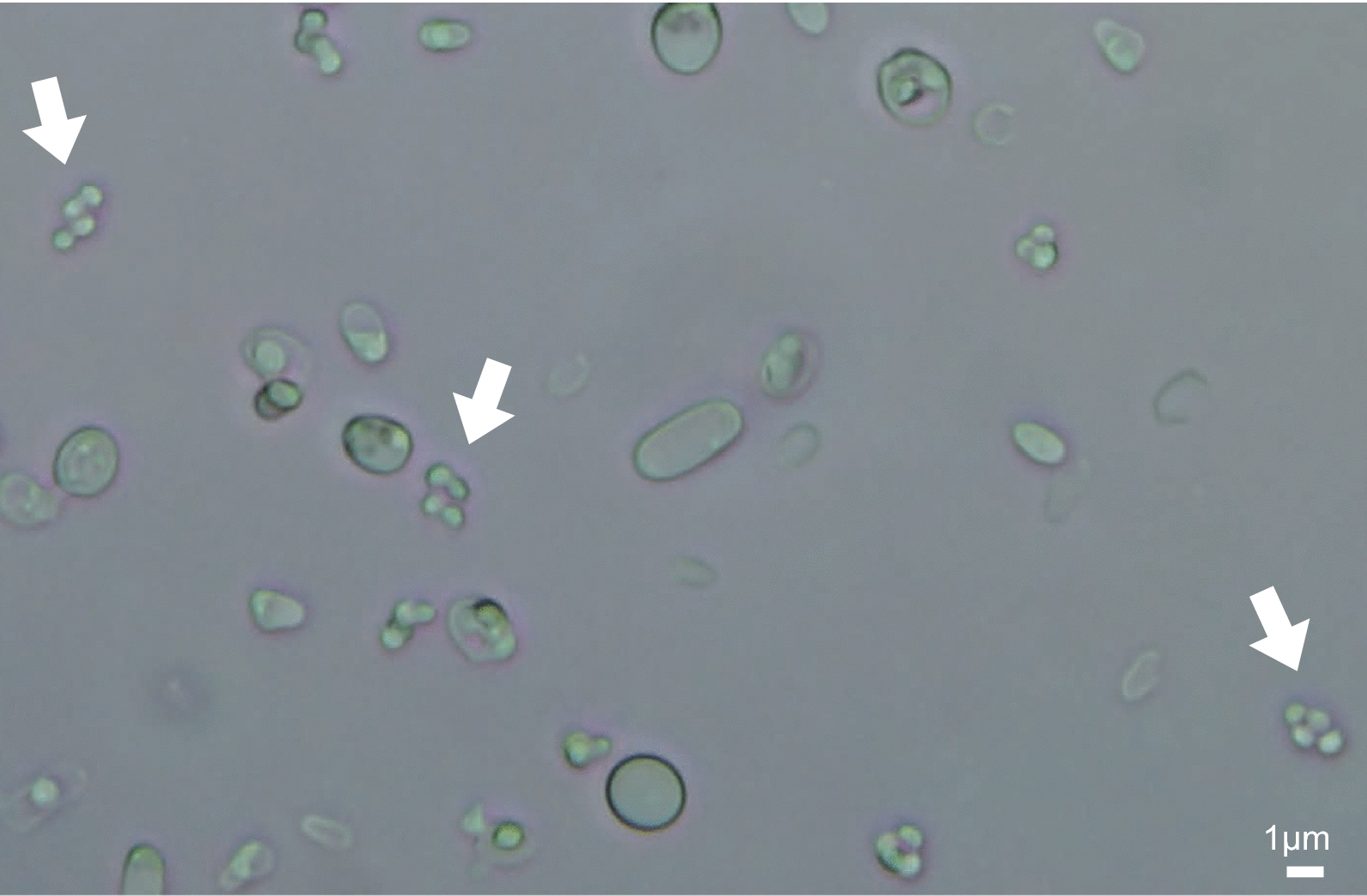
Bright field microscope image of a sporulated *K. phaffii* culture. Asci containing four spores are indicated with white arrows. Scale bar, 1 μm. This image is from a cross of CBS7435 × Pp2

Genetic crosses have previously been conducted in *K. phaffii*, but genetic analysis is difficult because its meiotic spores are much smaller than those of *S. cerevisiae*. *K.* *phaffii* makes asci containing four spores, but the asci are only 1–2 μm in diameter (Fig. 1), which is approximately four times smaller in *S. cerevisiae* [15, 16]. Moreover, the *K. phaffii* spores are difficult to separate by micromanipulation. They tend to stick together and form clumps, which can lead to mis-scoring of phenotypes if a clump of genetically heterogeneous spores germinates into a single colony [10]. For this reason, previous genetic analysis in *K.* *phaffii* has relied on random spore analysis methods, which are less powerful than the tetrad analysis commonly used in *S. cerevisiae* [10]. Similar problems make tetrad dissection in the related methylotrophic yeast *Ogataea polymorpha* difficult but not impossible [17, 18].

In this study, we sequenced the genomes of all the known natural isolates of *K. phaffii* to investigate its genetic diversity. We also made a cross between two isolates and sequenced the progeny, enabling the rate and distribution of meiotic recombination sites in *K. phaffii* to be investigated for the first time. We developed a method for tetrad analysis in *K. phaffii* that does not require asci to be dissected.

## Results

### Nucleotide diversity in natural isolates of *K. phaffii*

**Fig. 2:**
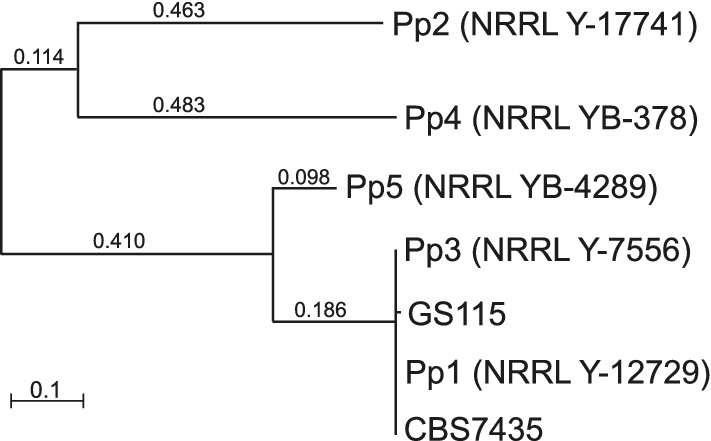
Phylogenetic tree of *K. phaffii* strains Pp1-Pp5, CBS7435 and GS115, based on 64,019 SNP sites. All non-trivial length branches have bootstrap values of 100%. Numbers on branches indicate branch lengths

We obtained five isolates of *K. phaffii* from the NRRL culture collection and sequenced their genomes. For convenience we refer to them as Pp1–Pp5; their strain numbers in the NRRL and CBS culture collections are given in Table 1. As far as we know, apart from CBS7435, no other isolates of this species are available from any public culture collections [5, 19]. We mapped the Illumina reads from each strain to the CBS7435 reference genome sequence [20] using BWA, and identified variant sites using the GATK SNP (single nucleotide polymorphism) calling pipeline. We included strain GS115 in this analysis, using the genome sequence reported by Love et al. [6]. A total of 64,019 variable SNP sites were identified among the six strains relative to CBS7435 (Table 1), and a phylogenetic tree of the strains was constructed from the variable sites (Fig. 2).

**Table 1:** *Komagataella phaffii* strains used in this study, and numbers of SNPs identified on each chromosome relative to CBS7435

Strain	NRRL strain number	CBS strain number	Source	SNP count relative to CBS7435					SNPs/kb
				Chr. 1	Chr. 2	Chr. 3	Chr. 4	Total	
Pp1	NRRL Y-12729	–	Unknown (received by NRRL from Guillermo Etienne Berumen, Instituto Mexicano del Petróleo)	2	0	1	0	3	0.00
Pp2	NRRL Y-17741	–	Emory oak (*Quercus emoryi*), Arizona, USA (W.T. Starmer)	12,330	11,279	11,295	8804	43,708	4.66
Pp3	NRRL Y-7556 (type strain)	CBS 2612 (type strain)	Black oak (*Quercus kelloggii*), California, USA (H.J. Phaff)	2	0	2	0	4	0.00
Pp4	NRRL YB-378	–	Elm (*Ulmus americana*), USA (L.J. Wickerham)	11,735	10,752	10,862	8489	41,838	4.46
Pp5	NRRL YB-4289	–	Black oak (*Quercus kelloggii*), California, USA (H.J. Phaff)	4188	4250	3486	3675	15,599	1.66
GS115	Derived from NRRL Y-11430 by mutagenesis (*his4*)	–	Commercial strain (Thermo Fisher Scientific)	21	12	22	14	69	0.01
CBS7435	NRRL Y-11430	CBS 7435	Genome reference strain. US patent granted to Phillips Petroleum.	n/a	n/a	n/a	n/a	n/a	n/a

NRRL, Northern Regional Research Laboratory, US Department of Agriculture; CBS, Centraalbureau voor Schimmelcultures (Westerdijk Institute), The Netherlands

Strains Pp3 and Pp1 were found to be essentially identical to CBS7435, differing from it by only 3 or 4 nucleotides in the 9.4 Mb nuclear genome. Pp3 (NRRL Y-7556/CBS2612) is a natural isolate from exudate of a black oak tree, and was designated by Kurtzman [19] as the type strain of *K. phaffii*. Pp1 and CBS7435, which were both deposited in culture collections by petroleum researchers (Table 1), seem to be duplicate accessions of the natural isolate Pp3. The mutagenized strain GS115 differs from its parent CBS7435 at 69 sites [6].

Among the three other natural isolates, Pp2 and Pp4 both show approximately 42,000–44,000 SNP differences from the CBS7435 reference, and Pp5 shows approximately 16,000 (Table 1). Pp2 and Pp4 are also quite divergent from each other (Fig. 2). The density of SNPs, at 1.66–4.66 SNPs per kb, is lower than the density seen in wild isolates of *S.* *cerevisiae* (e.g., the average nucleotide diversity among wild isolates from China is 8.08 differences/kb; [21]).

### Recovery of haploid segregants

We selected two of the most divergent strains from the phylogenetic tree, namely GS115 and Pp4, and crossed them in order to examine meiotic recombination in *K.* *phaffii.* GS115 has a *his4* mutation making it auxotrophic for histidine. We made a derivative of Pp4 that is auxotrophic for arginine (Pp4Arg^−^) by replacing the native *ARG4* gene with a *ble* cassette conferring resistance to zeocin (Zeo^R^). The *ARG4* and *HIS4* loci are both on *K. phaffii* chromosome 1, located 421 kb apart.

Diploid cells generated from the GS115 × Pp4Arg^−^ cross were identified by selecting for growth on media lacking His and Arg. Good mating was observed after incubating the cross for 3 days on diploid selection medium, resulting in a confluent patch of cells at the junction of streaked parental strains (Additional file 1: Fig. S1). The diploid strain was sporulated and tetrad formation was confirmed by microscopy, but we were unable to dissect the tetrads using a Singer Sporeplay dissection microscope.

**Fig. 3:**
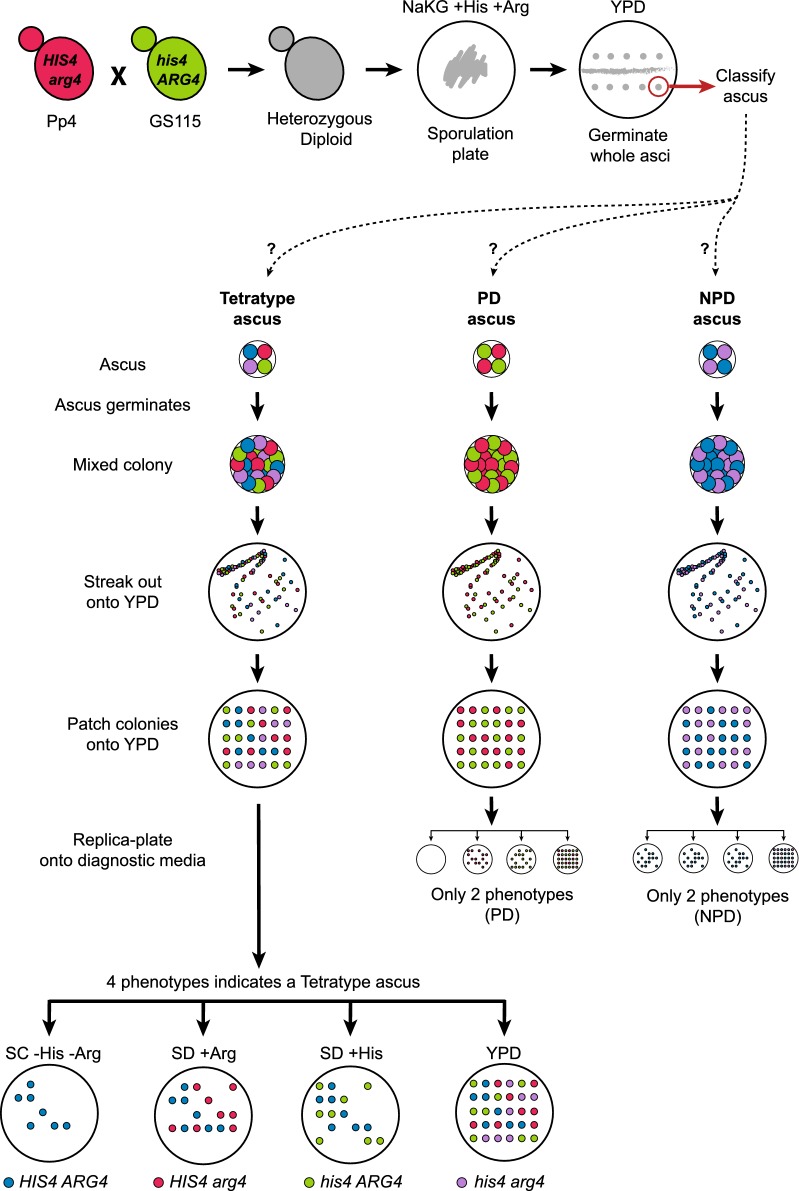
Experimental approach used to recover the four products of meiosis from an ascus without tetrad dissection. Parental strains Pp4 and GS115 were crossed to generate the resultant prototrophic heterozygous diploid. This diploid strain was sporulated under nitrogen-limiting conditions (NaKG + His + Arg medium). Whole asci containing four spores were picked up using a dissection microscope and placed on a YPD plate to germinate. The lower part of the diagram summarizes how asci were subsequently classified as tetratype, PD (parental ditype), or NPD (non-parental ditype) after streaking out and replica-plating. After germination, an ascus produces a mixed colony that contains a mixture of either 4 types of haploid cell (if the ascus is tetratype), or 2 types of haploid cell (if the ascus is PD or NPD), with respect to the *HIS4* and *ARG4* markers. After streaking out this mixed colony onto a fresh YPD plate and incubating for 2 days, single cells give rise to new colonies that each have a homogeneous genotype. These colonies are then patched in a grid pattern on another YPD plate, which is then replica-plated onto diagnostic media that allow the genotype of each colony to be inferred, hence enabling us to infer whether the ascus was tetratype, PD, or NPD. For asci inferred to be tetratype, the 4 types of haploid colonies (segregants) were retained for genome sequencing. For asci inferred to be PD or NPD, the segregants were discarded because it is not possible to identify all 4 products of the meiosis from their colony phenotypes. SC − His − Arg is synthetic complete media made without histidine and arginine. SD + Arg and SD + His are synthetic defined media supplemented with arginine or histidine. YPD is yeast peptone dextrose media (contains all amino acids)

Because *K. phaffii* cells do not mate on rich (YPD) media, we reasoned that if an ascus is placed intact on YPD so that its four spores germinate in situ, the resulting colony should contain a mixture of four different types of haploid cell that are the mitotic descendants of the four spores (Fig. 3). The four types of cell can be isolated simply by streaking out the original ascus-derived (mixed) colony, so that single cells initiate new colonies, each of which will have a homogeneous genotype that can be identified by replica plating onto appropriate media. Because our diploid was a double heterozygote (*HIS4/his4 ARG4/arg4*), it should produce three types of asci depending on how these markers segregate: parental ditypes (PD), non-parental ditypes (NPD), and tetratypes. In tetratype asci, which are formed if a single crossover (or any odd number of crossovers) occurs between the *HIS4* and *ARG4* loci, each spore has a different genotype and all four possible combinations of the two markers are present. Therefore, a tetratype ascus should produce four phenotypes after colonies are streaked out (His^+^ Arg^+^, His^−^ Arg^+^, His^+^ Arg^−^, and His^−^ Arg^−^), so we can identify colonies that are mitotic descendants of each of the four spores by replica plating onto appropriate media (Fig. 3). In contrast, PD and NPD asci both produce only two colony phenotypes, so they cannot be used to identify descendants of all four spores.

Following this logic, we isolated four-spored asci from the sporulated culture using the micromanipulator, and placed them, without dissection, onto YPD agar so that each ascus germinated into a colony. We then streaked out these colonies to obtain new colonies initiated by single cells, patched the new colonies onto fresh YPD, and replica-plated them to assess their His and Arg phenotypes, looking for tetratypes. In this manner, we successfully recovered the four meiotic products from five tetratype asci (Additional file 1: Fig. S1). Six other asci yielded three of the four expected phenotypes (‘trio’ asci), but we were unable to recover the fourth phenotype even after screening approximately 70 colonies from each of these asci. The missing phenotypes were His^−^ Arg^−^ in three trio asci, and His^+^ Arg^−^ in the other three trio asci (Additional file 2). The absence of one phenotype in the trios is possibly due to epistatic interactions between loci from the Pp4 and GS115 genetic backgrounds, in the particular combinations that were formed in some spores, resulting in the failure of one spore to germinate.

### High-resolution mapping of meiotic recombination in *K. phaffii*

We sequenced the genomes of 38 segregants: 4 segregants from each of 5 tetrads, and 3 segregants from each of 6 trios. Each genome was sequenced to approximately 100× Illumina coverage, and the reads were used to genotype each segregant at every SNP site between the Pp4 and CBS7435 reference genome (which is almost identical to GS115; see “Methods”). Data analysis was carried out on 41,838 SNP markers. The median distance between consecutive markers in our cross is 96 bp, which is comparable to the *S.* *cerevisiae* cross analyzed by Mancera et al. (~ 52,000 SNPs; median distance 78 bp) and the *Lachancea kluyveri* cross analyzed by Brion et al. (~ 57,000 markers used; median distance 196 bp) [22, 23].

**Fig. 4:**
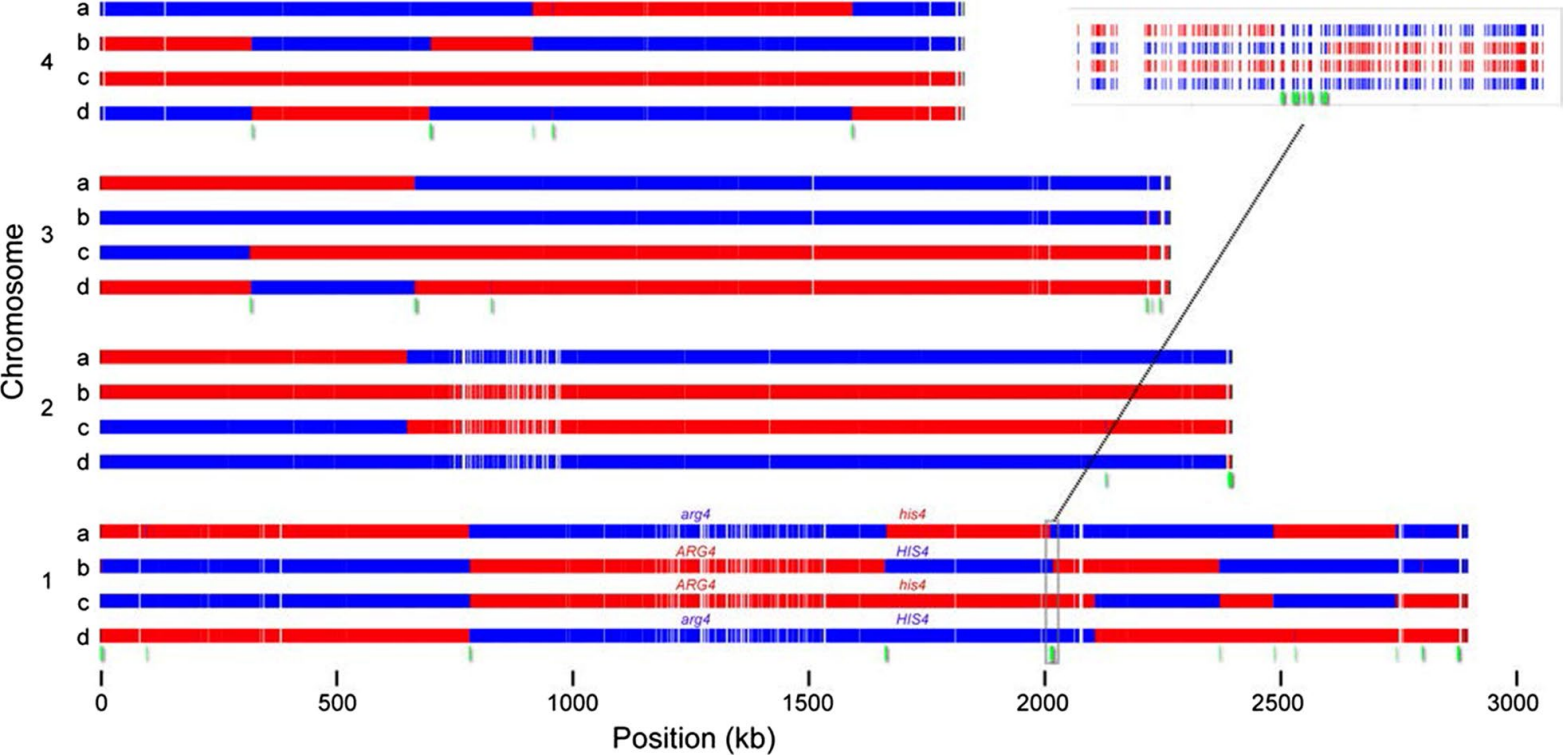
Segregation profile of one tetrad (Tetrad 1). Each group of four rows represents a single *K. phaffii* chromosome, indicated by numbers 1–4, and each row represents a spore, indicated by letters a-d. The four spores result from a single meiosis. Red segments represent regions inherited from the GS115 parent, and blue segments represent regions inherited from the Pp4 parent. *ARG4* and *HIS4* gene locations and genotypes are indicated. Red and blue bars are drawn at the location of every SNP difference between the two parental genomes. White gaps in the tracks are caused by stretches of DNA that are identical between the parents. Green segments below the chromosomes represent regions with non-Mendelian ratios (3:1 or 4:0) as illustrated in the inset

Figure 4 shows a graphical representation of the segregation of SNP alleles on the four chromosomes in one tetrad (Tetrad 1), which has a total of 15 crossover events. As expected, a single crossover occurred in the interval between the *ARG4* and *HIS4* loci, producing the four genotypes of the tetratype ascus. With these data we were able to determine the locations of crossovers, and to identify gene conversion tracts such as the region of 3:1 segregation shown in the inset in Fig. 4. Genotype calls from all tetrads and trios are given in Additional file 2, and plots of their segregation patterns similar to Fig. 4 are provided in Additional file 3.

We identified a total of 280 crossovers from the 11 meioses (Table 2). The mean number of crossovers per meiosis is 25.5, which is 3.5 times lower than the average in *S.* *cerevisiae* (90.5; [22]). The number varies about threefold (from 11 to 37) among the asci, with no systematic difference between tetrads and trios. The number of crossovers per meiosis in *K.* *phaffii* is also lower than in *S.* *paradoxus* (54.8), but similar to *Sch. pombe* (26.6) and *L.* *kluyveri* (19.9) [23–25].

**Table 2:** Summary of crossover (CO) counts in 11 *K. phaffii* asci

Ascus	Number of crossovers				
	Chr. 1	Chr. 2	Chr. 3	Chr. 4	Whole genome
*Tetrads*					
Tetrad 1	8	1	2	4	15
Tetrad 16	8	6	8	10	32
Tetrad 20	7	4	4	8	23
Tetrad 21	7	2	4	3	16
Tetrad 27	7	6	5	6	24
Total COs in 5 tetrads	37	19	23	31	110
COs per meiosis in 5 tetrads	7.4	3.8	4.6	6.2	22.0
*Trios*					
Trio 10	10	5	5	12	32
Trio 12	6	8	3	4	21
Trio 19	12	12	11	0	35
Trio 22	6	15	11	5	37
Trio 31	1	2	7	1	11
Trio 34	11	10	8	5	34
Total COs in 6 trios	46	52	45	27	170
*All 11 asci*					
Total COs in all 11 meioses	83	71	68	58	280
COs per meiosis in all 11	7.5	6.5	6.2	5.3	25.5
Chromosome length (kb)	2895.354	2396.458	2263.458	1827.941	9383.211
Crossovers/kb	0.00261	0.00269	0.00273	0.00288	0.00271
kb/crossover	383.7	371.3	366.1	346.7	368.6

We found on average one crossover per 369 kb across the genome, a number that is consistent among the four chromosomes (Table 2). The average number of crossovers per meiosis on each chromosome in our data has a linear correlation with chromosome size (Additional file 1: Fig. S2), in agreement with the pattern seen in a variety of other fungal genomes [23, 26–28]. The trend line for *K. phaffii* has an intercept of 1.37 crossovers, in agreement with the occurrence one obligatory crossover per chromosome, which follows from the essential role that crossovers play in chromosome segregation. Every chromosome sustained at least one crossover in every meiosis (Table 2), except for chromosome 4 in Trio 19 for which all spores were derived from the Pp4 parent (i.e. 3:1 or 4:0 segregation). The segregation pattern in Trio 19 possibly indicates a degree of meiotic dysfunction in *K. phaffii.*

### Recombination is suppressed in large regions around centromeres

**Fig. 5:**
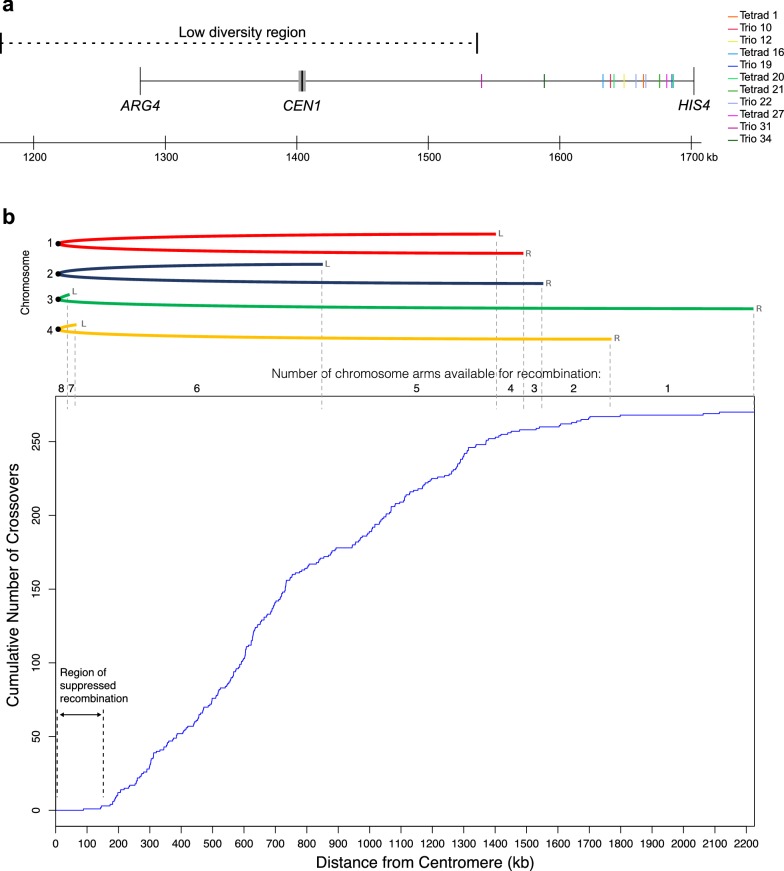
Suppressed recombination near *K. phaffii* centromeres. **a** Locations of crossovers in the *ARG4*–*HIS4* interval on chromosome 1. A crossover in this interval is obligatory in each of the 11 asci. Two asci (Tetrad 20 and Trio 22) had a second crossover in the interval, making 13 crossovers in total. The centromere (*CEN1*) is drawn to scale as a gray bar. The low diversity region around *CEN1* is indicated by a dashed line. **b** Distribution of distances from the centromere, for all 269 non-obligatory crossovers in our dataset. The graph shows the cumulative number of crossovers, plotted as a function of their distance from the centromere of the chromosome on which they occur. Data from all four chromosomes was pooled. The cartoon at the top shows the four chromosomes, folded at their centromeres, to show how many chromosome arms exist at any particular distance and hence are available for recombination (two of the eight chromosome arms are very short). Individual plots for each chromosome are shown in Additional file 1: Fig. S3. Obligatory crossovers in the *ARG4*–*HIS4* interval were excluded from the analysis in **b**. For Tetrad 20 and Trio 22, we arbitrarily designated the more centromere-proximal crossover as non-obligatory and the more distal one as obligatory

The *HIS4* and *ARG4* genes are both located on *K.* *phaffii* chromosome 1. They are 421 kb apart and on opposite sides of the centromere [20]. Our experimental design forced an obligatory crossover between these loci in every ascus. In addition to these obligatory crossovers in the 11 asci, two asci also had a second crossover in the *ARG4*–*HIS4* interval, involving a different pair of chromatids. The 13 crossovers are not randomly distributed along the 421-kb interval. Instead, they are all close to *HIS4* and they seem to avoid the centromere region (Fig. 5a); the hypothesis that crossovers are distributed uniformly in the interval is rejected by a statistical test (Kolmogorov–Smirnov test; *P* = 1.6e−6). The pattern suggests that crossing over near the centromere is suppressed.

To investigate whether other crossovers are also suppressed near centromeres, we plotted the locations of all 269 non-obligatory crossovers in our dataset as a function of their distance from centromeres, both for the whole genome (Fig. 5b), and for each chromosome individually (Additional file 1: Fig. S3). The plots confirm that the recombination rate is low near centromeres. The distribution has an inflection point at a distance of approximately 150–200 kb from the centromere (Fig. 5b), with only 3 crossovers occurring < 150 kb from the centromere (the closest one is 88 kb away). Suppressed recombination around the centromere is seen on all four chromosomes (Additional file 1: Fig. S3). *K. phaffii* has two metacentric chromosomes (chrs. 1 and 2), and two acrocentric chromosomes (chrs. 3 and 4) (Fig. 5b and [29]). For the two metacentric chromosomes, suppression on both arms creates a zone of approximately 300 kb with low recombination.

### Low diversity in large regions around *CEN1* and *CEN2*

We found that the regions of suppressed recombination around the centromeres of chromosomes 1 and 2 coincide with regions of low sequence diversity among *K.* *phaffii* isolates. The low diversity regions are visible in Fig. 4 as areas with many white gaps in the red and blue tracks, near the centers of chromosomes 1 and 2. The white gaps indicate places where there are no SNP differences between the parental strains GS115 and Pp4, i.e. they have identical sequences.

**Fig. 6:**
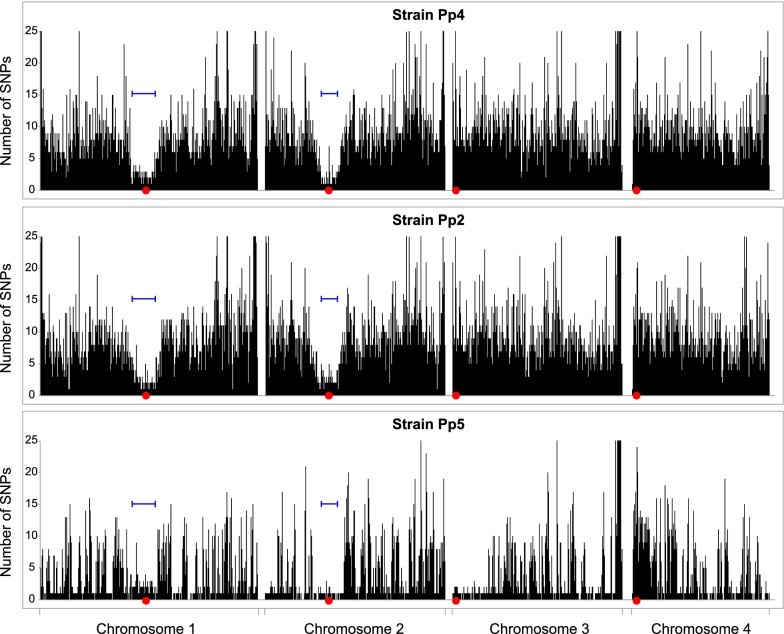
SNP density variation in natural isolates. For strains Pp4, Pp2, and Pp5, the number of SNP differences from CBS7435 in each 1-kb interval along each chromosome is plotted. Red dots mark centromeres, and blue bars mark the locations of the low-diversity regions that were defined in Pp4. The Y-axis maximum has been truncated to 25 SNPs/kb

To examine this pattern in more detail, we plotted SNP diversity in the three natural isolates Pp4, Pp2 and Pp5, in 1-kb bins, relative to the CBS7435 reference genome (Fig. 6). Pp4 and Pp2 both show large regions of low diversity around *CEN1* and *CEN2*, but not around the other two centromeres. The pattern in Pp5 is consistent, though this strain also shows some other large regions with high similarity to CBS7435 (such as the left end of chr. 3) which possibly indicate shared ancestry between Pp5 and Pp3/CBS7435 because these strains came from the same source (Table 1). We defined low-diversity regions around *CEN1* and *CEN2* of Pp4 as the regions in which the average number of SNPs was less than 1/kb, compared to a whole genome average of 4.5/kb. The lengths of the low-diversity regions are 362 kb around *CEN1* and 240 kb around *CEN2* (Fig. 6).

The low-diversity regions around *CEN1* and *CEN2* coincide approximately with their regions of suppressed recombination, but they are more than 20 times larger than the centromeres themselves. *K. phaffii* centromeres have a simple inverted repeat (IR) structure, with a total size of approximately 6 kb, and are located in non-transcribed regions of 6–9 kb that are flanked by protein-coding genes [29]. In contrast, the low-diversity regions in Pp4 and Pp2 extend outwards from the centromere for more than 100 kb in each direction (230 kb to the left and 132 kb to the right at *CEN1*; 110 kb to the left and 130 kb to the right at *CEN2*). There are many protein-coding genes in the low-diversity regions (including *ARG4*) and the gene density is the same as in the rest of the genome. All the crossovers in the *ARG4*–*HIS4* interval occurred outside the low-diversity region around *CEN1* (Fig. 5a).

## Discussion

We found that *K. phaffii* has lower genetic diversity than *S.* *cerevisiae*, and that only four distinct natural isolates of this species are available in culture collections. We demonstrated that tetrad analysis is possible, though cumbersome, in this species and found that its meiotic recombination rate is approximately 3.5 times lower than in *S.* *cerevisiae*, which is one of the most recombinogenic species known. Compared to other yeasts, the *S. cerevisiae* genome is organized into chromosomes that are shorter and more numerous, as a result of its whole-genome duplication. When chromosome size is taken into account, the numbers of crossovers per megabase per meiosis in *K.* *phaffii* are similar to the numbers in *L.* *kluyveri* and *Sch.* *pombe* (Additional file 1: Fig. S2), but substantially lower than in *S. cerevisiae*.

The most surprising result from our analysis was the discovery of a very large region of suppression of meiotic recombination around all four *K. phaffii* centromeres. Recombination is also suppressed close to centromeres in *S. cerevisiae*, but the effect only extends over approximately 10 kb [22, 27, 31], compared to approximately 300 kb in *K. phaffii*. In *S.* *cerevisiae*, crossovers near centromeres are suppressed by the kinetochore, which attaches to a single nucleosome that contains centromeric histone H3 (Cse4), and by cohesin bound to the pericentromeric region [31–33]. At *K.* *phaffii* centromeres, the region of Cse4 binding is less than 10 kb long [29], so the reason why recombination is suppressed over a 300 kb region is unclear, but possibly related to a structural difference between IR centromeres (*K. phaffii*) and point centromeres (*S. cerevisiae*).

On the two metacentric chromosomes (chrs. 1 and 2), the region of low recombination around the centromere coincides with a region of low sequence diversity. This low diversity around *K. phaffii* centromeres contrasts with the high variation reported in centromeric regions of the fission yeast *Sch. pombe* [30], but it should be noted that the fission yeast high-diversity regions include extensive arrays of centromeric tandem repeats, whereas the *K.* *phaffii* low-diversity regions are largely non-repetitive. The pattern on *K. phaffii* chromosomes 1 and 2 is consistent with previous observations of low sequence diversity in non-recombining regions of animal [34, 35] and plant [36] genomes, which has been attributed to background selection. In background selection, when deleterious mutations arising in regions of low recombination are eliminated by natural selection, any linked neutral variants are also eliminated and hence genomic diversity in the region is reduced [37]. In general, genomic diversity is positively correlated with the recombination rate among regions of eukaryotic genomes, though this pattern is only weakly seen in *S. cerevisiae* [38, 39]. In contrast to the metacentric chromosomes, the centromere regions of the acrocentric *K.* *phaffii* chromosomes (chrs. 3 and 4) show low recombination without low sequence diversity. Chromosome 4 may be a special case, because *CEN4* is located approximately in the center of a 138-kb region that is inverted between cells of mating types a and α, so the region is in opposite orientations on the two copies of chromosome 4 in diploid cells. A single crossover near *CEN4* would generate chromosomes with huge deletions or duplications [12, 29], making the cells inviable, and we did not see any crossovers in the 138-kb region. Nevertheless, nucleotide diversity in this region is high.

Our work provides a foundation for future quantitative trait locus analysis in *K. phaffii*, by quantifying the rate of meiotic recombination in this species. Because most *K. phaffii* strains used in biotechnology are derivatives of the single isolate CBS7435, it may be possible to identify beneficial alleles in other isolates and introduce them into commercial strains, either by crossing or by genome editing. However, we also found that the pool of potential alleles available for strain improvement is very small, compared for example to *S. cerevisiae*, because only four natural isolates of *K.* *phaffii* are currently known. Therefore, the discovery of additional isolates should be a priority for the future improvement of *K. phaffii* as a host for recombinant protein expression.

## Methods

### Strain generation and growth conditions

The NRRL strains of *K. phaffii* used in this study (Table 1) were obtained from the USDA NRRL Culture Collection (USA). Strain CBS7435 was obtained from the Spanish Type Culture Collection as CECT 11047. All strains were grown in YPD (1% yeast extract, 2% peptone, 2% glucose) medium at 30 °C and 200 rpm of agitation.

Parental strains used to generate the recombination map were the laboratory strain GS115 (Thermo Fisher Scientific) and the natural isolate Pp4 (NRRL YB-378). GS115 is auxotrophic for histidine (His^−^) due to a C557R point mutation in *HIS4* [6, 8, 40], and we confirmed this by resequencing the GS115 genome. Pp4 was made auxotrophic for arginine (Arg^−^) by replacing *ARG4* [41] with the drug marker *ble* conferring zeocin resistance. The pILV5-*ble* cassette (ZEO) from *K. phaffii* plasmid pJ902-15 (Atum, USA) was flanked by 1 kb of right- (HR) and 1 kb of left- (HL) homology arms originating from the sequences flanking the *ARG4* gene of Pp4 (Additional file 4). These three fragments were PCR-amplified individually (New England Biolabs Q5 high-fidelity 2× master mix, 56 °C annealing temperature, 25 cycles) and joined in a single step by overlap extension PCR (1st step: equimolar mix of PCR fragments ZEO, HR and HL, NEB Q5 high-fidelity 2× master mix, 60 °C annealing temperature, 12 cycles, no primers added; 2nd step: addition of primers for fragment HL + ZEO + HR, 67 °C annealing temperature, 25 cycles) to produce a 3.5 kb insert flanked by *Sac*I restriction sites. The HL + ZEO + HR insert was ligated into the unique SacI site in plasmid pUC57 (Thermo Fisher Scientific) and transformed into One Shot TOP10 Chemically Competent *E. coli* cells (Thermo Fisher Scientific) generating the 6.25 kb ARG4del plasmid (Additional file 4). This plasmid was digested with the restriction enzyme pair BseRI–AflII for homologous recombination into Pp4, replacing the native *ARG4* gene with the ZEO construct. Transformation was carried out by electroporation following Atum’s guidelines, and transformant colonies harboring the *ARG4* deletion (*ARG4*∆::*ble*) were selected on YPD plates supplemented with zeocin (200 μg ml^−1^). The Pp4Arg^−^ mutant phenotype was confirmed by absence of growth in Synthetic Defined (SD) minimal medium (2% glucose, 6.7 g l^−1^ yeast nitrogen base without amino acids) and by PCR amplification of the targeted locus.

The cross of parental strains GS115 and Pp4Arg^−^ was carried out by making parallel streaks of the two parental strains on YPD, and then velvet replica plating these streaks onto a mating plate twice at right angles so that they intersected as a grid [10]. The mating plate contained NaKG agar media (0.5% sodium acetate, 1% potassium chloride, 1% glucose, 2% bacto agar) plates supplemented with l-histidine (50 mg l^−1^) and l-arginine (100 mg l^−1^) (NaKG + His + Arg). The mating plate was incubated for 2 days at room temperature and subsequently replica plated onto diploid selection medium (SC − His − Arg: SD medium supplemented with − Arg/− His drop-out mix). Diploids were incubated for 3 days at 30 °C, and streaked for a second time for phenotype confirmation and generation of single colonies.

### Recovery of asci and haploid segregants

Sporulation was induced by streaking diploid cells on NaKG + His + Arg plates and incubating for ≥ 5 days at room temperature. A loop of sporulated material was resuspended in sterile distilled water and digested for 10 min at 30 °C with Zymolyase 100T (Stratech). The mixture was washed and resuspended in sterile distilled water, and a 10 μl drop of this suspension was left to run across a YPD plate, forming a horizontal streak. The plate was incubated for 4 h at 30 °C to allow asci to swell and become more visible under the 40× lens of a SporePlay + dissection microscope (Singer Instruments). Whole asci were picked up and placed individually on the YPD plate, which was incubated for 2 days at 30 °C until colonies were visible. Each colony starting from a single ascus was picked and streaked onto a fresh YPD plate for single colonies (segregants). Approximately 20 to 70 segregants per ascus were picked and patched on a fresh YPD plate, incubated at 30 °C overnight, and replica plated onto a set of four diagnostic plates for identification of each segregant type (Fig. 3; Additional file 1: Fig. S1). The diagnostic plates contained: SC − His − Arg media (only *HIS4 ARG4* segregants grow); SD + His media (*his4 ARG4* and *HIS4 ARG4* segregants grow); SD + Arg media (*HIS4 arg4* and *HIS4 ARG4* segregants grow); and YPD (*his4 arg4* and all other segregants grow). By visual inspection and comparison of the colonies that grew on each media it was possible to identify the 4 (or 3) meiotic products originating from tetratype asci. Asci that yielded only two phenotypes were considered to be PD or NPD and discarded. It is important to note that *K. phaffii* spores tend to clump together, which can be noticed by the high number of colonies showing prototrophic phenotypes (Additional file 1: Fig. S1; [10]). Therefore segregants from all candidate tetratype asci were re-patched onto all diagnostic media to confirm their phenotypes before genome sequencing.

### Genomic DNA extraction and genome sequencing

Genomic DNA was extracted from stationary-phase cultures by homogenization with glass beads followed by phenol–chloroform extraction and ethanol precipitation. Purified DNA was concentrated with the Genomic DNA Clean & Concentrator-10 (Zymo Research). Natural isolates Pp1–Pp5 were sequenced at the University of Missouri core facility using an Illumina HiSeq 2500 instrument, with single-end 51 bp reads, to approximately 30× coverage, and assembled using SPAdes version 3.10 [42]. The parental strains used in the cross (Pp4Arg^−^ and GS115) and the 38 segregants, were each sequenced to approximately 100× coverage (paired-end 150 bp reads) on an Illumina HiSeq 4000 by BGI Tech Solutions (Hong Kong).

### SNP calling

BAM alignments of Illumina reads from each segregant to the CBS7435 reference genome [20] were generated using the Burrows-Wheeler Aligner (BWA) with default parameters [43]. Unmapped reads were removed using SAMtools [44] and headers were added using the AddOrReplaceReadGroups program in Picard Tools [http://picard.sourceforge.net]. Variants against the reference were called with the GATK HaplotypeCaller tool in DISCOVERY genotyping mode with the following parameters: “-stand_emit_conf 10 -stand_call_conf 30 –min_base_quality_score 20 emitRefConfidence GVCF”. For the segregants from each tetrad and trio these GVCF files were then combined using GATK into joint genotype calls for each tetrad/trio. For improved clarity in downstream analysis the 69 SNPs between CBS7435 and GS115 [6] were removed from these lists—they are an artefact of our decision to use CBS7435 as the reference genome for alignment (because it had better assembly and annotation), whereas GS115 was the actual parent in our cross.

### Crossover detection

A bespoke Java program was used, taking the joint genotype calls as input, to list the genotype of the 4 segregants in each tetrad (or 3 segregants in each trio) at every SNP site (Additional file 2). Genotypes of segregants at SNP sites were coded as 0 (for a nucleotide matching GS115) or 1 (for a nucleotide matching Pp4). Sites called as indels or heterozygous were discarded. Genotype lists for each tetrad were input into the plotting tool in the ReCombine package [45] to make red/blue maps of the segregation patterns in asci (Fig. 4; Additional file 3), after modifying the tool to plot 4 chromosomes instead of 16. Crossover and noncrossover events were identified using a program to find every SNP site in a trio/tetrad whose segregation pattern was different from neighboring site(s), and scoring manually. We ignored noncrossovers involving only a single SNP site, and ignored the subtelomeric regions of each chromosome (Chr. 1: coordinates < 8 kb and > 2861 kb; Chr. 2: < 12 kb and > 2373 kb; Chr. 3: > 2168 kb; Chr. 4: < 2 kb and > 1795 kb). For analysis of recombination rates near centromeres, we calculated distances from centromeres as the distance from the midpoint of the unique central region between the IRs: coordinates 1,404,539 (*CEN1*), 847,809 (*CEN2*), 37,576 (*CEN3*), and 61,908 (*CEN4*) in the reference genome sequence of Sturmberger et al. [20].

### Phylogenetic tree

The phylogenetic tree of strains CBS7435, GS115 and Pp1–Pp5 was generated using PhyML (HKY model) [46] from the 64,019 SNP sites that vary among these strains, calculated using GATK (as described above for the segregants) for Pp1–Pp5 compared to CBS7435, and using MUMmer’s *nucmer* whole genome alignment program [47] for the GS115 to CBS7435 comparison.

## Supplementary information

**Additional file 1.** Additional figures and legends supporting the results described in text.

**Additional file 2.** Genotype calls at SNP sites in each ascus. Values of “0″ indicate the same nucleotide as in GS115, and “1” indicates a match to Pp4. Spores were designated as types **a** (*his4 arg4*), **b** (*HIS4 ARG4*), **c** (*his4 ARG4*) or **d** (*HIS4 arg4*). All four types of spore were recovered from tetrads; Trios 10, 22 and 19 lack a spore of type **a**, and Trios 31, 34 and 12 lack a spore of type **d**. Chromosome coordinates are from the CBS7435 reference genome assembly of Sturmberger et al. [20], NCBI accession numbers LT962476.1 to LT962479.1.

**Additional file 3.** Segregation profiles of all *K. phaffii* tetrads and trios analyzed, as in Fig. 4.

**Additional file 4.** Nucleotide sequence of the ARG4del plasmid used to delete *ARG4* in strain Pp4.

## Data Availability

Strains and plasmids are available upon request. The datasets supporting the conclusions of this article are included within the article and its additional files. Genome sequence data have been submitted to the NCBI database (BioProject number PRJNA592463).
