# Supplementary material for: Genomic diversity and meiotic recombination among isolates of the biotech yeast Komagataella phaffii (Pichia pastoris)
Source: Microb Cell Fact. 2019 Dec 4;18:211. doi: 10.1186/s12934-019-1260-4 (PMC6894112; doi:10.1186/s12934-019-1260-4)
Supplement: Supplementary file 1 — Additional file 1. Additional figures and legends supporting the results described in text. [file 12934_2019_1260_MOESM1_ESM.pdf]

Figure S1. Crossing of parental strains and identification of segregant genotypes. (a) Crossing of parental strains. Parallel streaks of the two parental strains Pp4Arg$^-$ and GS115 were made on a YPD plate, and then replica-plated twice perpendicularly onto a mating plate (NaKG +His +Arg) to make a grid pattern. After growth, the mating plate was then replica plated onto the SC -His -Arg selection plate shown. Diploid cells (His$^+$ Arg$^+$) grow at the intersections of the streaks. **(b)** Initial identification of segregants from Tetrad 27, by replica plating onto 4 media. An example of each class of segregant is circled. **(c)** Verification of segregant genotypes from Tetrad 27 by a second round of replica plating.

Figure S2. Relationship between chromosome size and the average number of crossovers per meiosis on that chromosome, in four ascomycete yeast species: *K. phaffii* (this study), *L. kluyveri* [23], *S. cerevisiae* [22], and *Sch. pombe* [25]. For *Sch. pombe*, we considered only the 10 F1 segregants in [25], omitting F2 segregants, and assumed that the actual number of crossovers in the ascus was twice the number of crossovers detected in the single segregant that was sequenced from each ascus; hence the results for *Sch. pombe* differ from [23]. The number of crossovers on the largest *Sch. pombe* chromosome may be abnormally low due to a large inversion [25]. For *L. kluyveri*, chromosome C has the lowest number of crossovers, and its low rate has been attributed to the origin of most of this chromosome from an ancient introgression event [23].

Figure S3. Locations of crossovers on each chromosome, as a function of their distance from the centromere. Red lines and numbers 1-2 indicate the number of chromosome arms that exist at any particular distance. All chromosomes are plotted on the same horizontal scale. Obligatory crossovers in the *ARG4-HIS4* interval on chromosome 1 were excluded from this analysis (for Tetrad 22 and Trio 20, which have two crossovers in this interval, we arbitrarily designated the more centromere-proximal crossover as non-obligatory and the more distal one as obligatory).

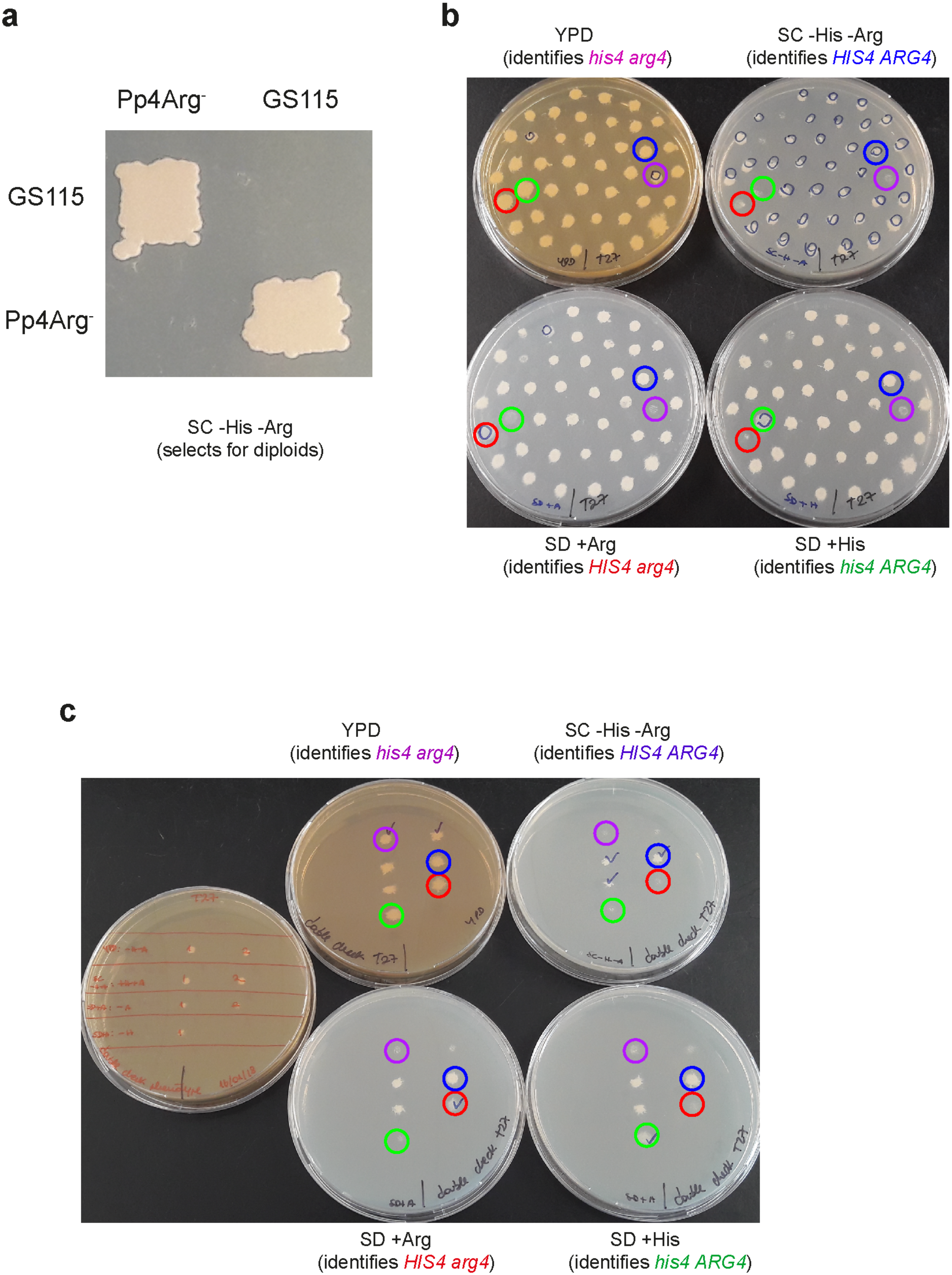
a
Pp4Arg-
GS115
GS115
Pp4Arg-
SC -His -Arg
(selects for diploids)
b
YPD
(identifies his4 arg4)
SC -His -Arg
(identifies HIS4 ARG4)
SD +Arg
(identifies HIS4 arg4)
SD +His
(identifies his4 ARG4)
c
YPD
(identifies his4 arg4)
SC -His -Arg
(identifies HIS4 ARG4)
SD +Arg
(identifies HIS4 arg4)
SD +His
(identifies his4 ARG4)

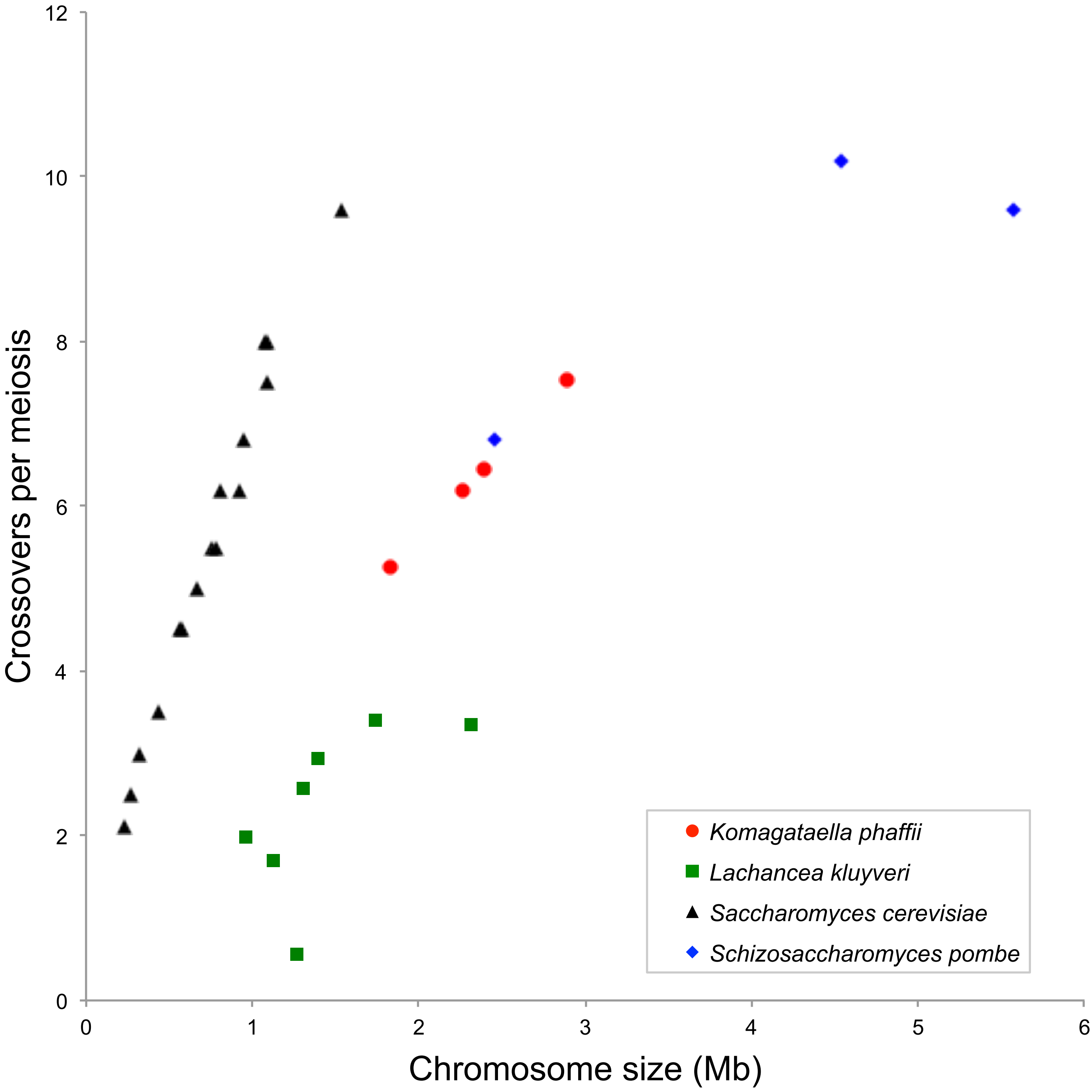

Crossovers per meiosis
12
10
8
6
4
2
0
0
1
2
3
4
5
6
Chromosome size (Mb)
Komagataella phaffii
Lachancea kluyveri
Saccharomyces cerevisiae
Schizosaccharomyces pombe

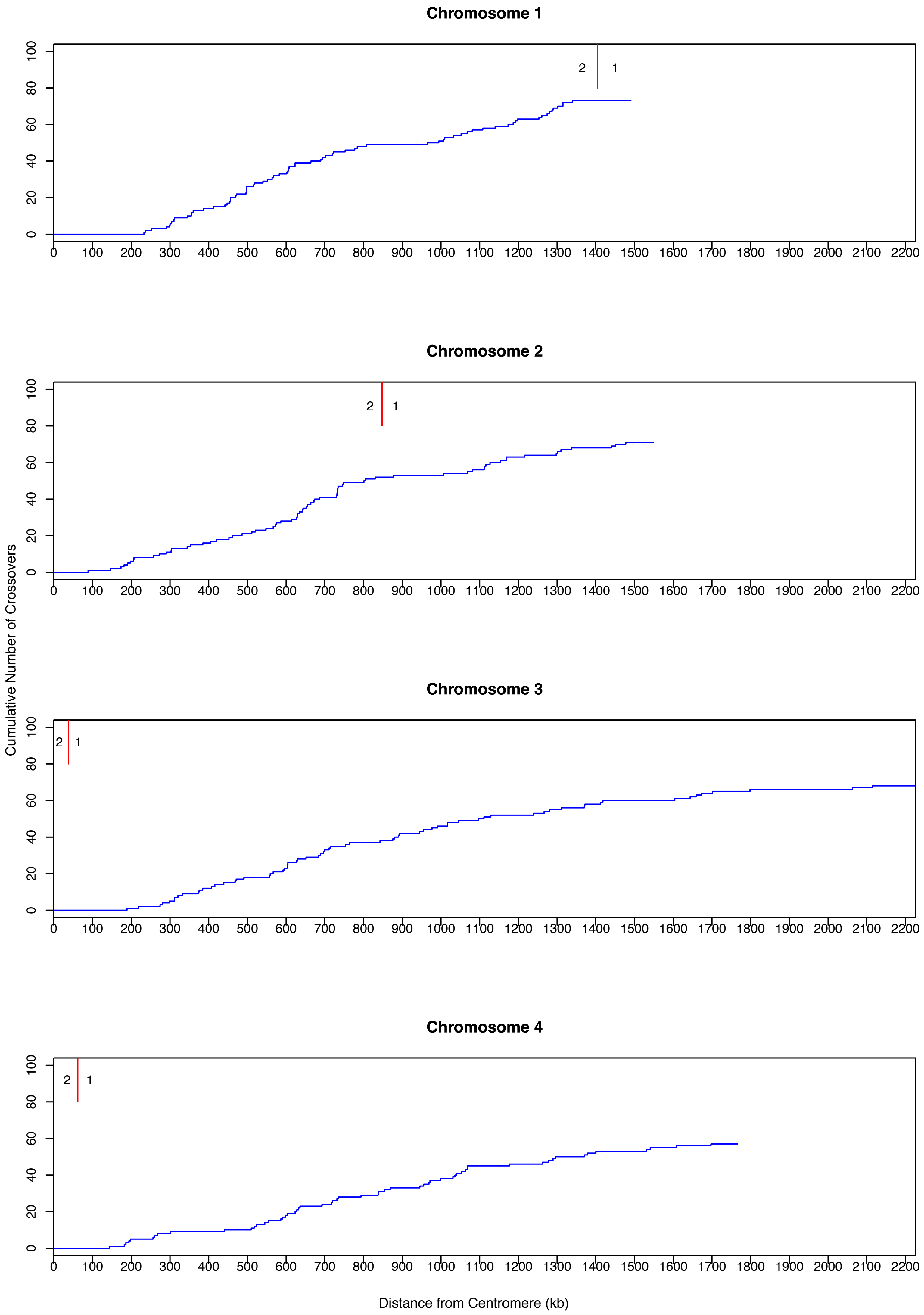
Chromosome 1
Chromosome 2
Chromosome 3
Chromosome 4
2
1
Cumulative Number of Crossovers
0
20
40
60
80
100
0
100
200
300
400
500
600
700
800
900
1000
1100
1200
1300
1400
1500
1600
1700
1800
1900
2000
2100
2200
Distance from Centromere (kb)
