# Supplementary figures and images for: Genomic diversity and meiotic recombination among isolates of the biotech yeast Komagataella phaffii (Pichia pastoris)
Source: Microb Cell Fact. 2019 Dec 4;18:211. doi: 10.1186/s12934-019-1260-4 (PMC6894112; doi:10.1186/s12934-019-1260-4)

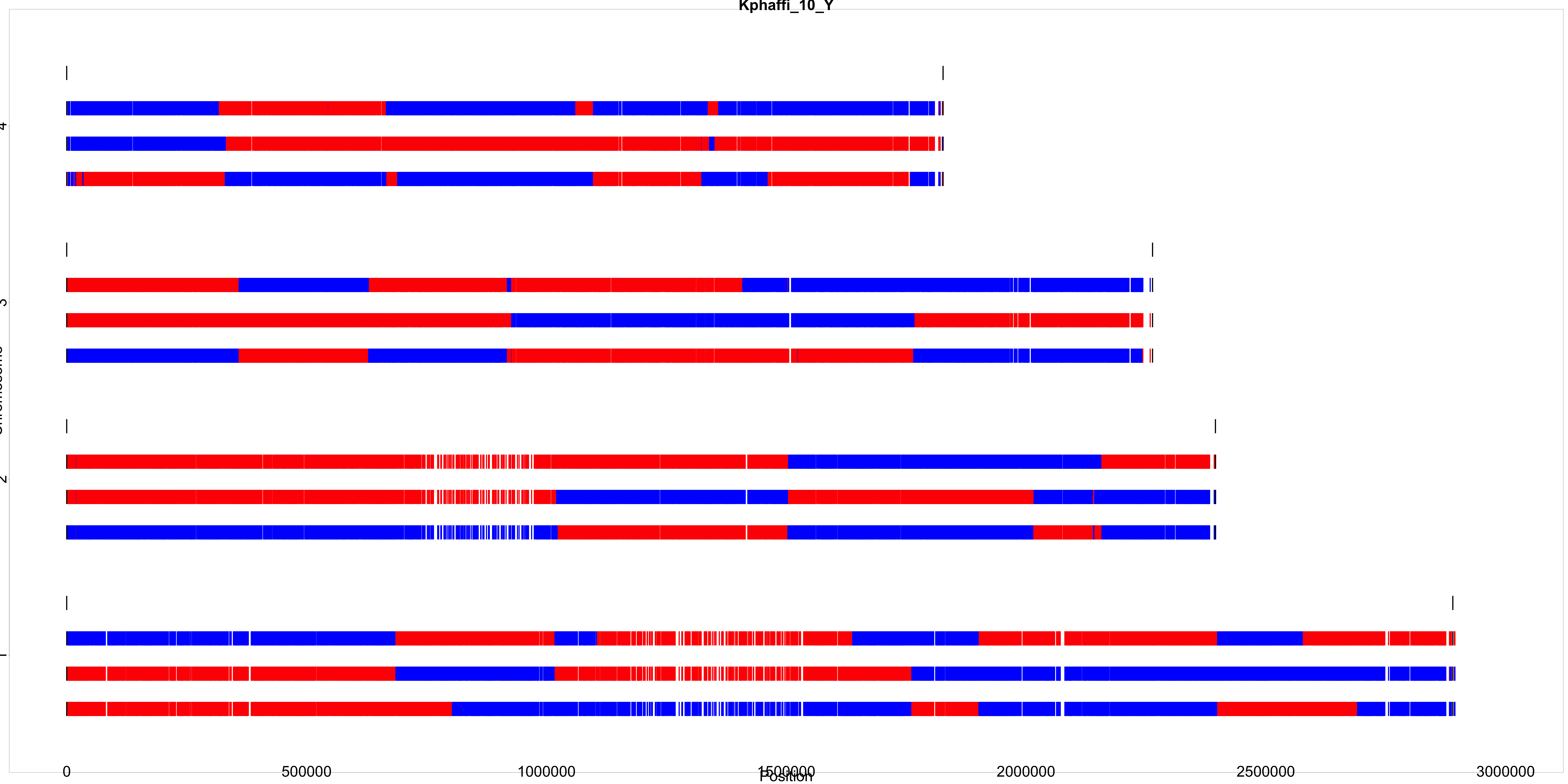

Supplement: Supplementary file 3 — Additional file 3. Segregation profiles of all K. phaffii tetrads and trios analyzed, as in Fig. 4. [file 12934_2019_1260_MOESM3_ESM.zip › Kphaffi_10_Y.png]

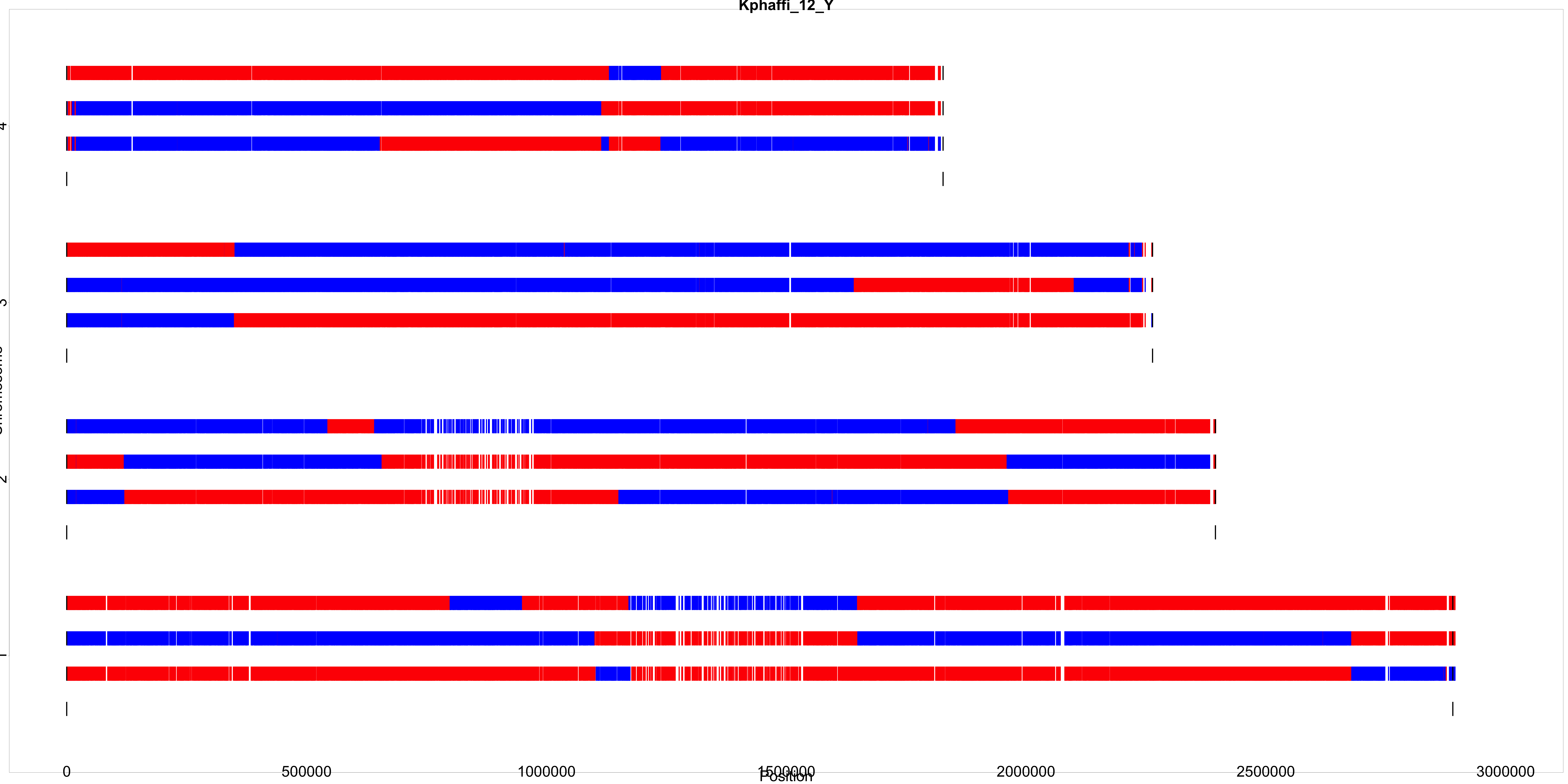

Supplement: Supplementary file 3 — Additional file 3. Segregation profiles of all K. phaffii tetrads and trios analyzed, as in Fig. 4. [file 12934_2019_1260_MOESM3_ESM.zip › Kphaffi_12_Y.png]

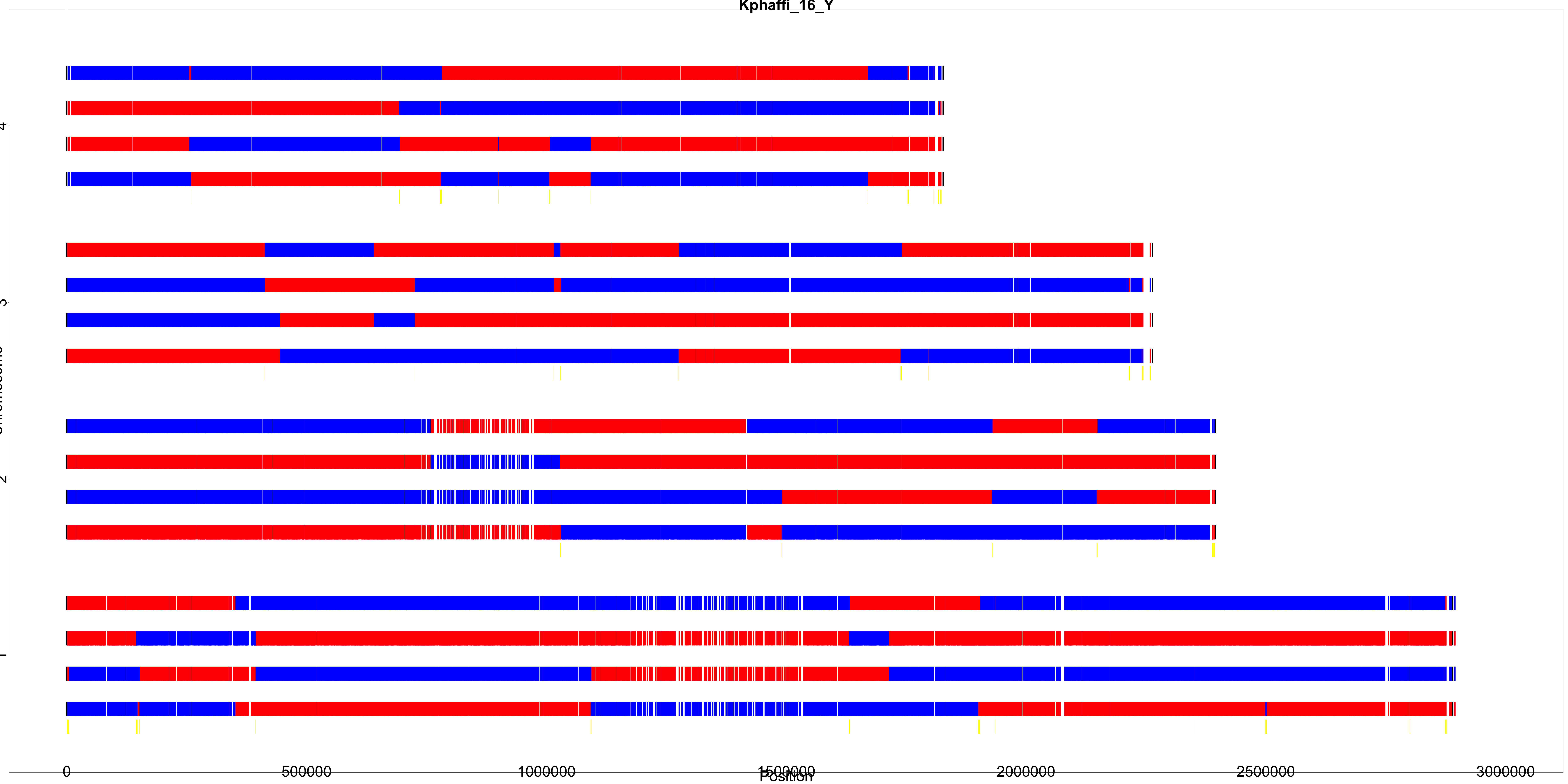

Supplement: Supplementary file 3 — Additional file 3. Segregation profiles of all K. phaffii tetrads and trios analyzed, as in Fig. 4. [file 12934_2019_1260_MOESM3_ESM.zip › Kphaffi_16_Y.png]

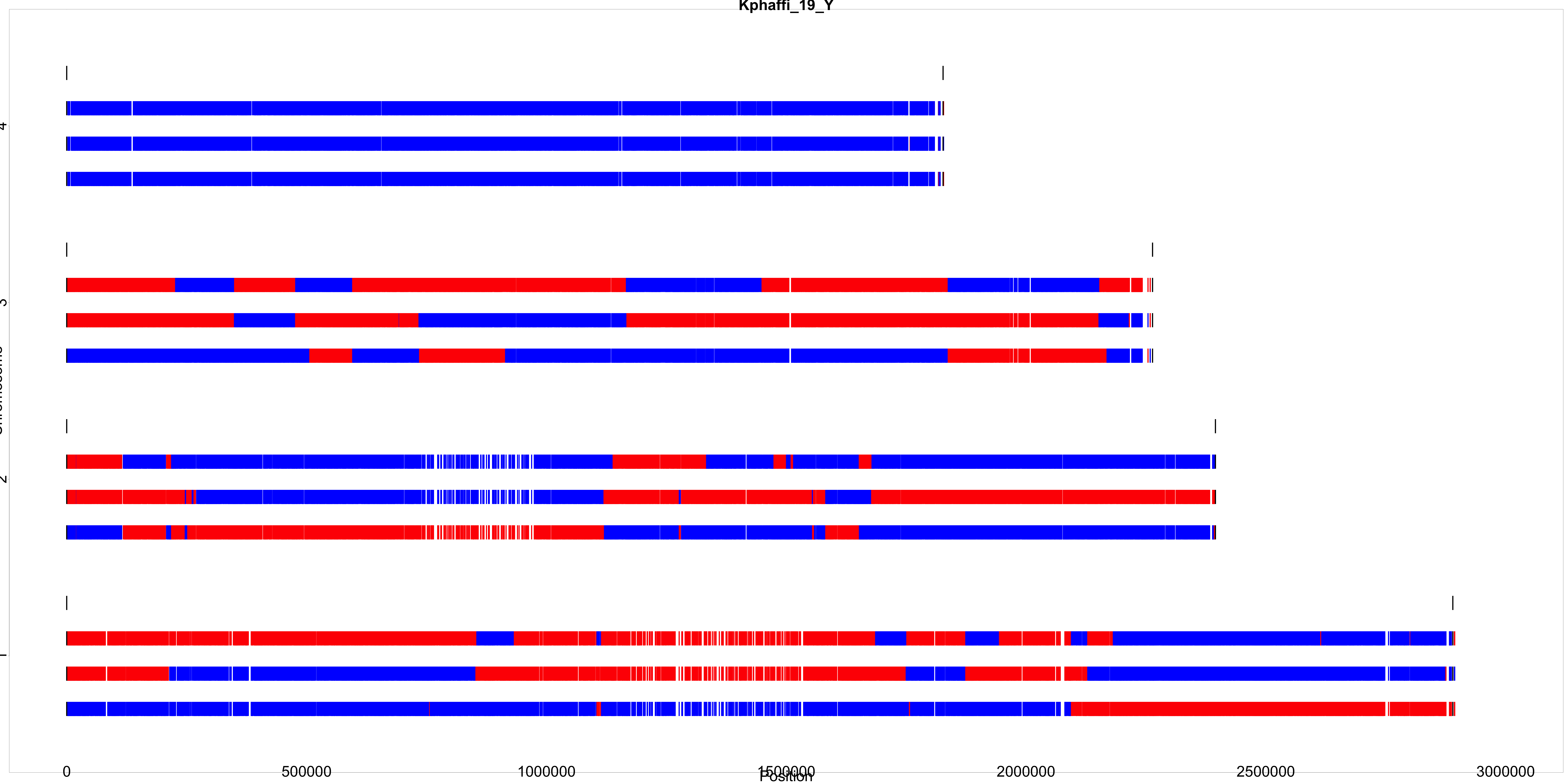

Supplement: Supplementary file 3 — Additional file 3. Segregation profiles of all K. phaffii tetrads and trios analyzed, as in Fig. 4. [file 12934_2019_1260_MOESM3_ESM.zip › Kphaffi_19_Y.png]

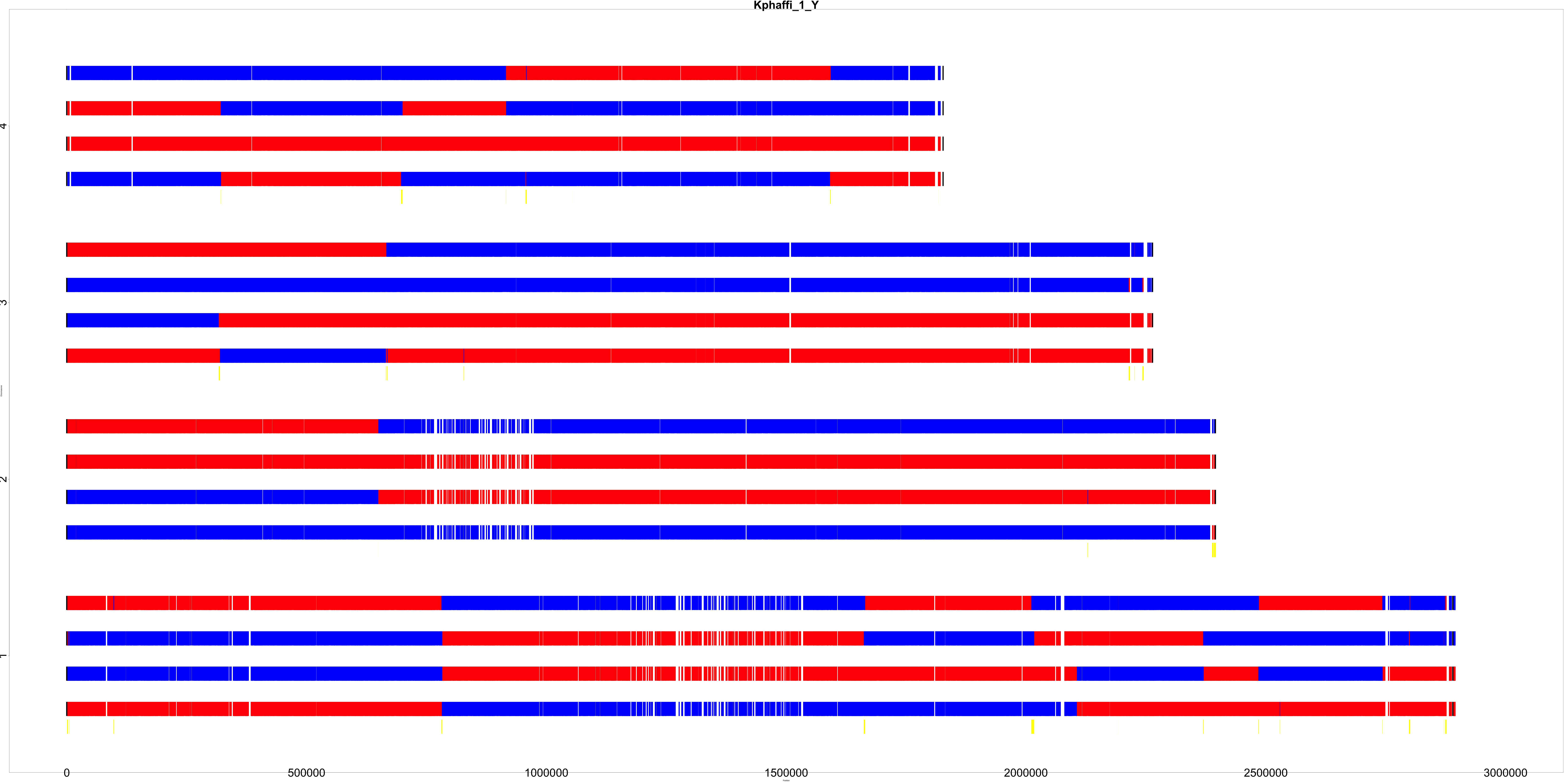

Supplement: Supplementary file 3 — Additional file 3. Segregation profiles of all K. phaffii tetrads and trios analyzed, as in Fig. 4. [file 12934_2019_1260_MOESM3_ESM.zip › Kphaffi_1_Y.png]

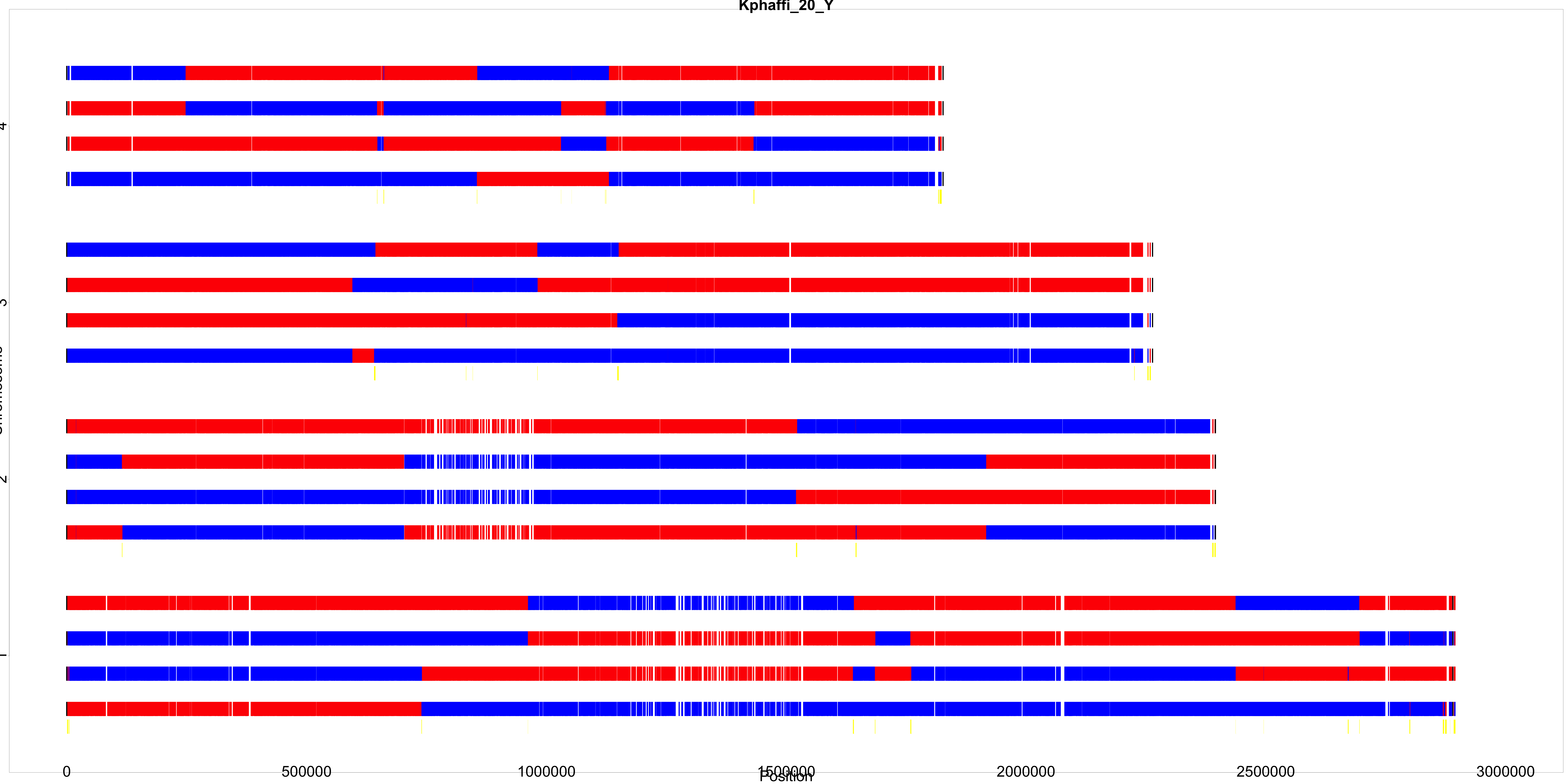

Supplement: Supplementary file 3 — Additional file 3. Segregation profiles of all K. phaffii tetrads and trios analyzed, as in Fig. 4. [file 12934_2019_1260_MOESM3_ESM.zip › Kphaffi_20_Y.png]

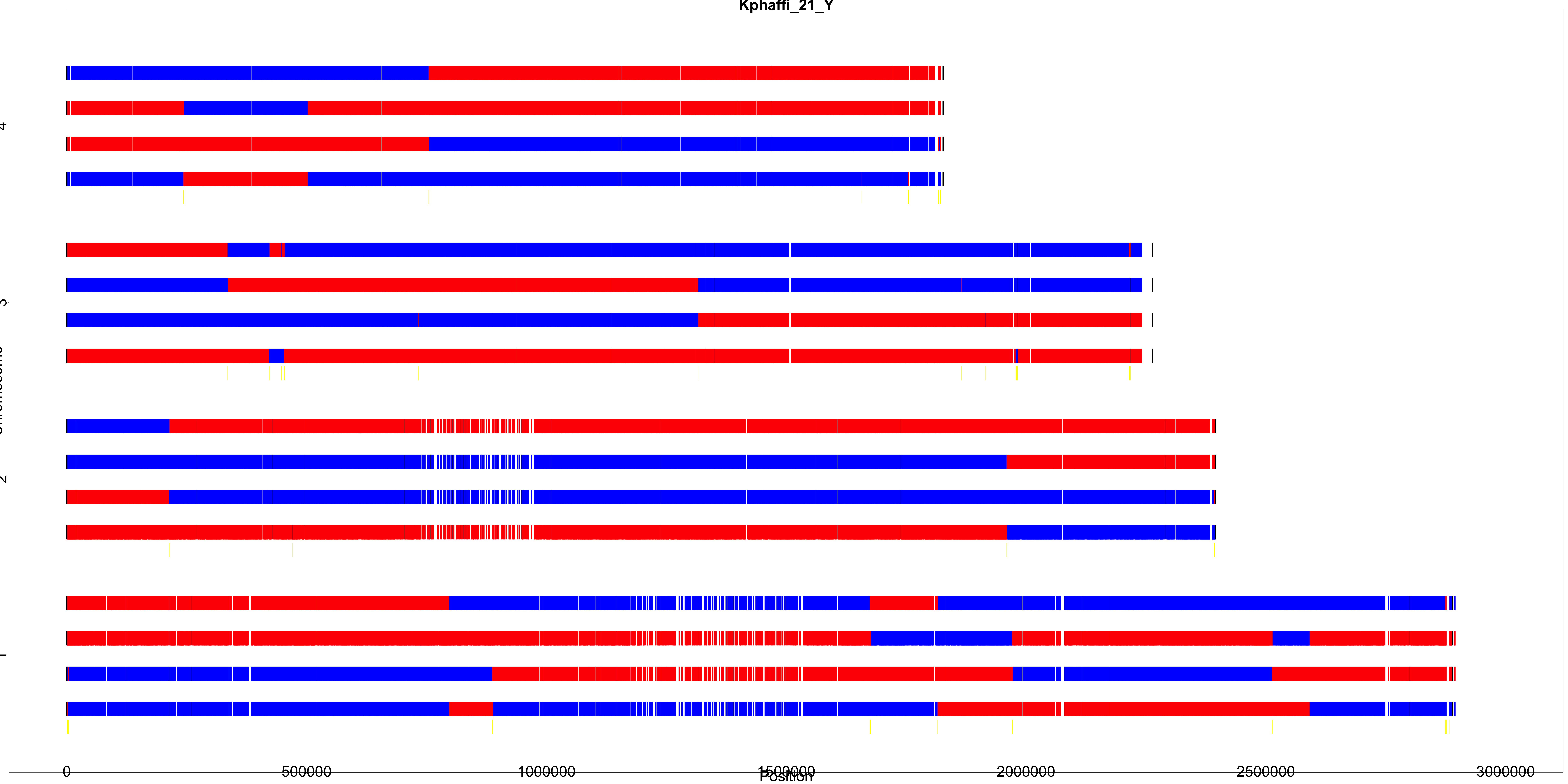

Supplement: Supplementary file 3 — Additional file 3. Segregation profiles of all K. phaffii tetrads and trios analyzed, as in Fig. 4. [file 12934_2019_1260_MOESM3_ESM.zip › Kphaffi_21_Y.png]

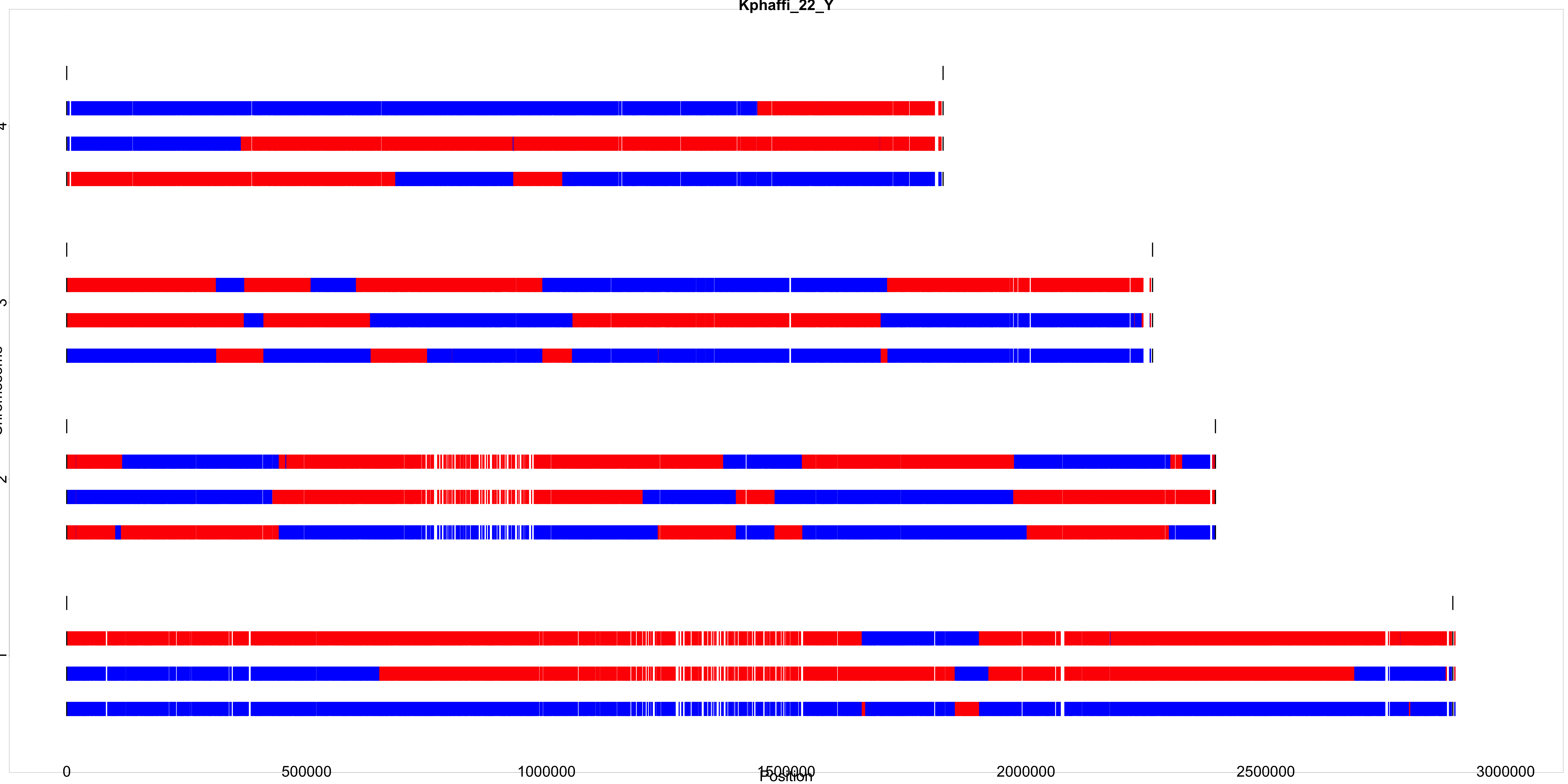

Supplement: Supplementary file 3 — Additional file 3. Segregation profiles of all K. phaffii tetrads and trios analyzed, as in Fig. 4. [file 12934_2019_1260_MOESM3_ESM.zip › Kphaffi_22_Y.png]

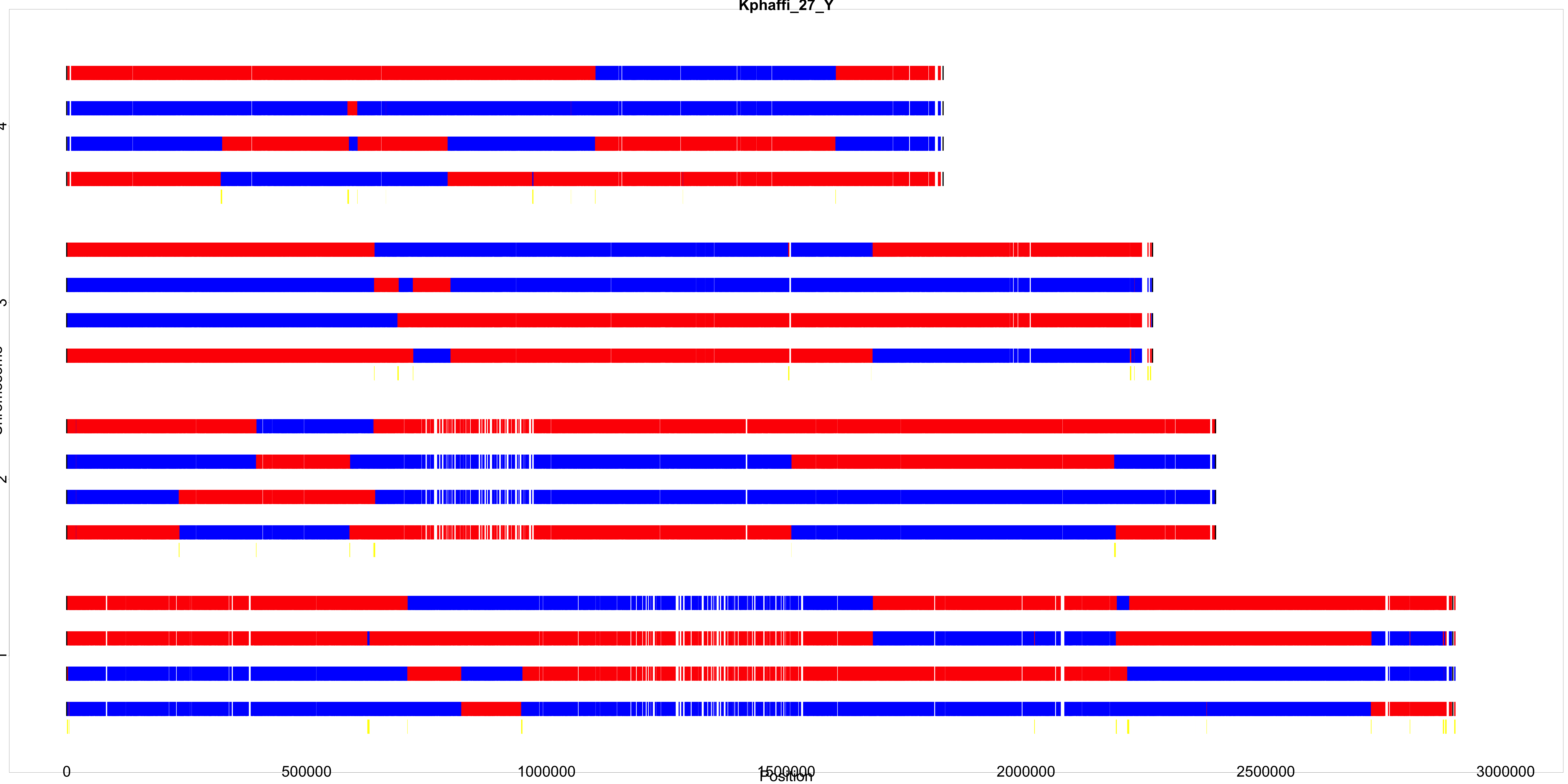

Supplement: Supplementary file 3 — Additional file 3. Segregation profiles of all K. phaffii tetrads and trios analyzed, as in Fig. 4. [file 12934_2019_1260_MOESM3_ESM.zip › Kphaffi_27_Y.png]

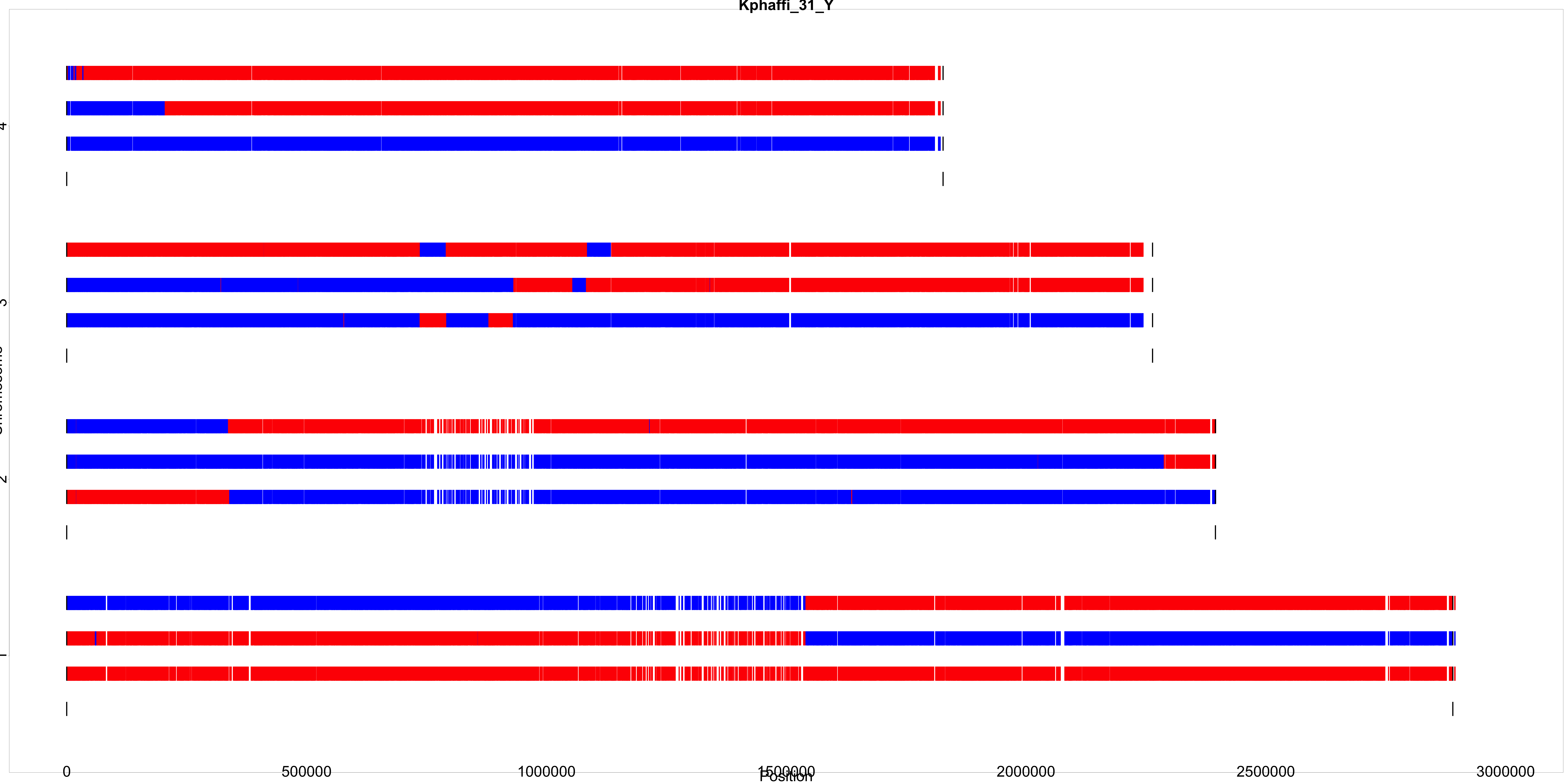

Supplement: Supplementary file 3 — Additional file 3. Segregation profiles of all K. phaffii tetrads and trios analyzed, as in Fig. 4. [file 12934_2019_1260_MOESM3_ESM.zip › Kphaffi_31_Y.png]

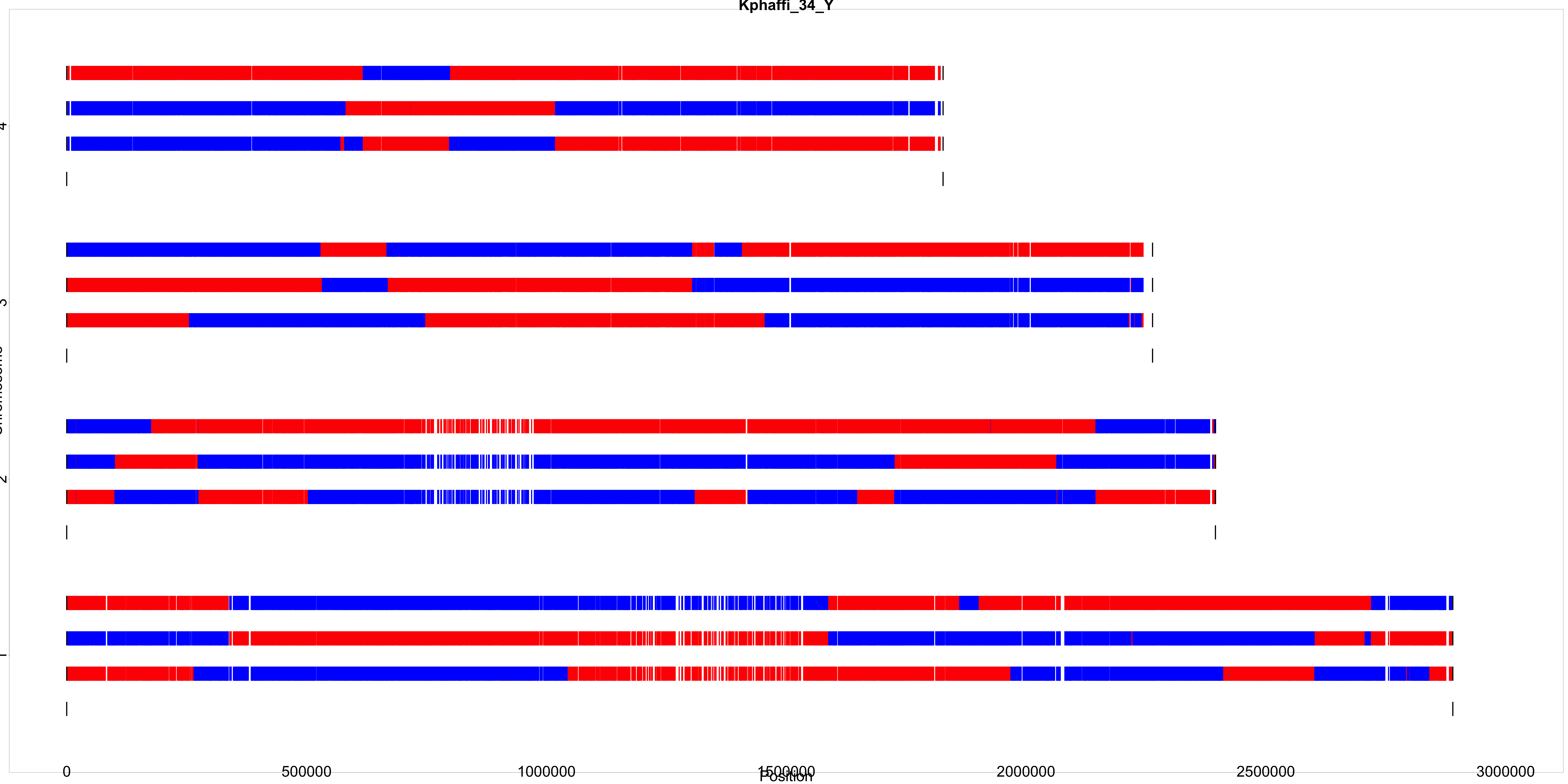

Supplement: Supplementary file 3 — Additional file 3. Segregation profiles of all K. phaffii tetrads and trios analyzed, as in Fig. 4. [file 12934_2019_1260_MOESM3_ESM.zip › Kphaffi_34_Y.png]
